# Significance Analysis of Prognostic Signatures

**DOI:** 10.1371/journal.pcbi.1002875

**Published:** 2013-01-24

**Authors:** Andrew H. Beck, Nicholas W. Knoblauch, Marco M. Hefti, Jennifer Kaplan, Stuart J. Schnitt, Aedin C. Culhane, Markus S. Schroeder, Thomas Risch, John Quackenbush, Benjamin Haibe-Kains

**Affiliations:** 1 Department of Pathology, Beth Israel Deaconess Medical Center and Harvard Medical School, Boston, Massachusetts, United States of America; 2 Biostatistics and Computational Biology, Dana-Farber Cancer Institute, Boston, Massachusetts, United States of America; 3 Biostatistics, Harvard School of Public Health, Boston, Massachusetts, United States of America; 4 Cancer Biology, Dana-Farber Cancer Institute, Boston, Massachusetts, United States of America; 5 Bioinformatics and Computational Genomics Laboratory, Institut de Recherches Cliniques de Montréal (IRCM), Montreal, Quebec, Canada; National University of Singapore, Singapore

## Abstract

A major goal in translational cancer research is to identify biological signatures driving cancer progression and metastasis. A common technique applied in genomics research is to cluster patients using gene expression data from a candidate prognostic gene set, and if the resulting clusters show statistically significant outcome stratification, to associate the gene set with prognosis, suggesting its biological and clinical importance. Recent work has questioned the validity of this approach by showing in several breast cancer data sets that “random” gene sets tend to cluster patients into prognostically variable subgroups. This work suggests that new rigorous statistical methods are needed to identify biologically informative prognostic gene sets. To address this problem, we developed Significance Analysis of Prognostic Signatures (SAPS) which integrates standard prognostic tests with a new prognostic significance test based on stratifying patients into prognostic subtypes with random gene sets. SAPS ensures that a significant gene set is not only able to stratify patients into prognostically variable groups, but is also enriched for genes showing strong univariate associations with patient prognosis, and performs significantly better than random gene sets. We use SAPS to perform a large meta-analysis (the largest completed to date) of prognostic pathways in breast and ovarian cancer and their molecular subtypes. Our analyses show that only a small subset of the gene sets found statistically significant using standard measures achieve significance by SAPS. We identify new prognostic signatures in breast and ovarian cancer and their corresponding molecular subtypes, and we show that prognostic signatures in ER negative breast cancer are more similar to prognostic signatures in ovarian cancer than to prognostic signatures in ER positive breast cancer. SAPS is a powerful new method for deriving robust prognostic biological signatures from clinically annotated genomic datasets.

## Author Summary

A major goal in biomedical research is to identify sets of genes (or “biological signatures”) associated with patient survival, as these genes could be targeted to aid in diagnosing and treating disease. A major challenge in using prognostic associations to identify biologically informative signatures is that in some diseases, “random” gene sets are associated with prognosis. To address this problem, we developed a new method called “Significance Analysis of Prognostic Signatures” (or “SAPS”) for the identification of biologically informative gene sets associated with patient survival. To test the effectiveness of SAPS, we use SAPS to perform a subtype-specific meta-analysis of prognostic signatures in large breast and ovarian cancer meta-data sets. This analysis represents the largest of its kind ever performed. Our analyses show that only a small subset of the gene sets found statistically significant using standard measures achieve significance by SAPS. We identify new prognostic signatures in breast and ovarian cancer and their corresponding molecular subtypes, and we demonstrate a striking similarity between prognostic pathways in ER negative breast cancer and ovarian cancer, suggesting new shared therapeutic targets for these aggressive malignancies. SAPS is a powerful new method for deriving robust prognostic biological pathways from clinically annotated genomic datasets.

## Introduction

The identification of pathways that predict prognosis in cancer is important for enhancing our understanding of the biology of cancer progression and for identifying new therapeutic targets. There are three widely-recognized breast cancer molecular subtypes, “luminal” (ER+/HER2−) [1], [2], [3], [4], “HER2-enriched” (HER2+) [5], [6] and “basal-like” (ER−/HER2−) [6], [7], [8], [9] and a considerable body of work has focused on defining prognostic signatures in these [10], [11]. Several groups have analyzed prognostic biological pathways across breast cancer molecular subtypes [12], [13], [14]; a tacit assumption is that if a gene signature is associated with prognosis, it is likely to encode a biological signature driving carcinogenesis.

Recent work by Venet et al. has questioned the validity of this assumption by showing that most random gene sets are able to separate breast cancer cases into groups exhibiting significant survival differences [15]. This suggests that it is not valid to infer the biologic significance of a gene set in breast cancer based on its association with breast cancer prognosis and further, that new rigorous statistical methods are needed to identify biologically informative prognostic pathways.

To this end, we developed Significance Analysis of Prognostic Signatures (SAPS). The score derived from SAPS summarizes three distinct significance tests related to a candidate gene set's association with patient prognosis. The statistical significance of the *SAPS_score_* is estimated using an empirical permutation-based procedure to estimate the proportion of random gene sets achieving at least as significant a SAPS score as the candidate prognostic gene set. We apply SAPS to a large breast cancer meta-dataset and identify prognostic genes sets in breast cancer overall, as well as within breast cancer molecular subtypes. Only a small subset of gene sets that achieve statistical significance using standard statistical measures achieves significance using SAPS. Further, the gene sets identified by SAPS provide new insight into the mechanisms driving breast cancer development and progression.

To assess the generalizability of SAPS, we apply it to a large ovarian cancer meta-dataset and identify significant prognostic gene sets. Lastly, we compare prognostic gene sets in breast and ovarian cancer molecular subtypes, identifying a core set of shared biological signatures driving prognosis in ER+ breast cancer molecular subtypes, a distinct core set of signatures associated with prognosis in ER− breast cancer and ovarian cancer molecular subtypes, and a set of signatures associated with improved prognosis across breast and ovarian cancer.

## Results

### Significance Analysis of Prognostic Signatures (SAPS)

The assumption behind SAPS is that to use a prognostic association to indicate the biological significance of a gene set, a gene set should achieve three distinct and complimentary objectives. First, the gene set should cluster patients into groups that show survival differences. Second, the gene set should perform significantly better than random gene sets at this task, and third, the gene set should be enriched for genes that show strong univariate associations with prognosis.

**Figure 1:**
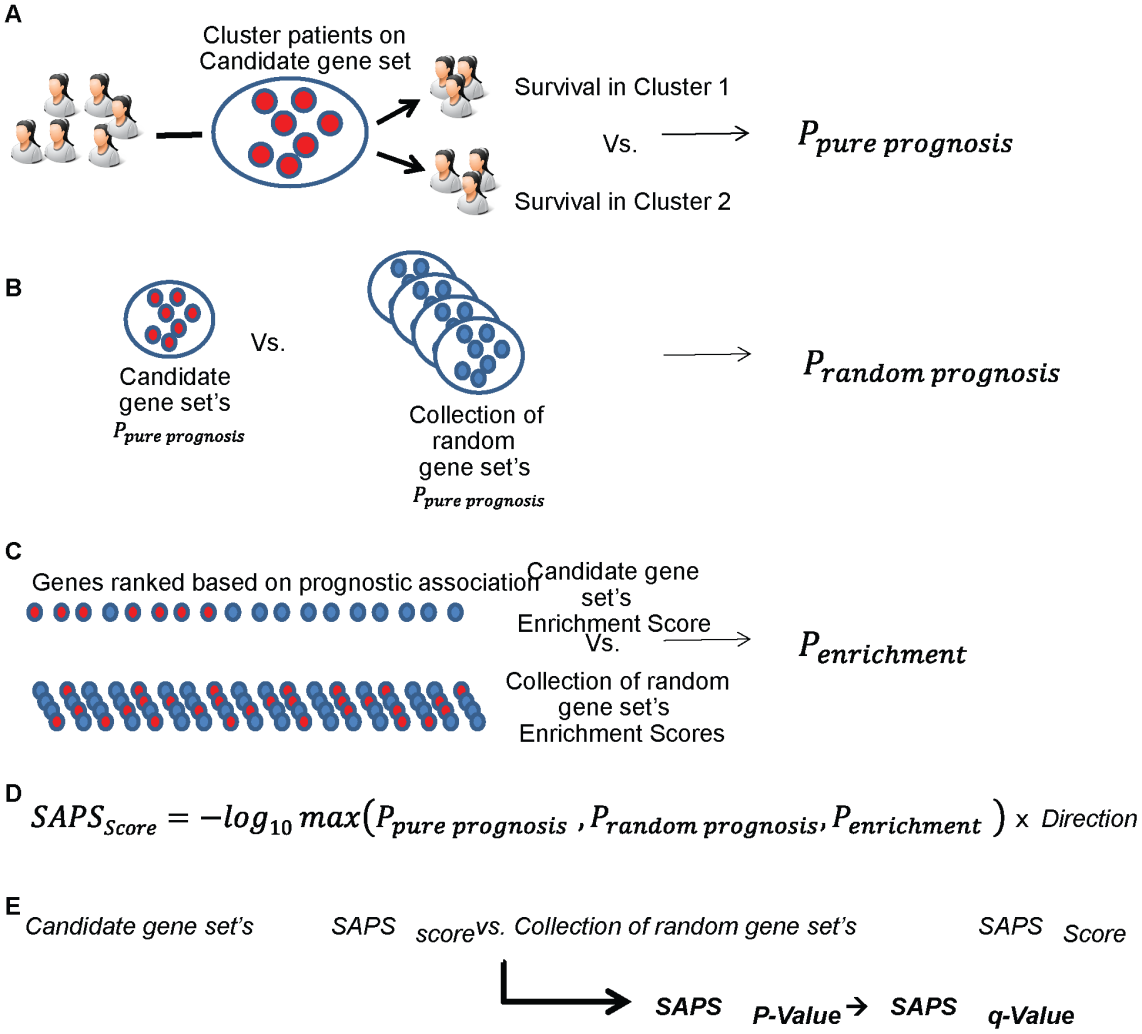
Overview of SAPS method. The SAPS method computes three P values for a candidate gene set (A–C). These P values are summarized in the *SAPS_score_* (D) and statistical significance of a *SAPS_score_* is estimated by permutation testing (E).

To achieve this end, SAPS computes three p-values (*P_pure_*, *P_random_*, and *P_enrichment_*) for a candidate prognostic gene set. These individual *P-Values* are summarized in the *SAPS_score_*. The statistical significance of the *SAPS_score_* is estimated by permutation testing involving permuting the gene labels (**Figure 1**).

To compute the *P_pure_*, we stratify patients into two groups by performing k-means clustering (k = 2) of an *n×p* data matrix, consisting of the *n* patients in the dataset and the *p* genes in the candidate prognostic gene set. We then compute a log-rank *P-Value* to indicate the probability that the two groups of patients show no survival difference (**Figure 1A**).

Next, we assess the probability that a random gene set would perform as well as the candidate gene set in clustering cases into prognostically variable groups. This *P-Value* is the *P_random_*. To compute the *P_random_*, we randomly sample genes to create random gene sets of similar size to the candidate gene set. We randomly sample *r* gene sets, and for each random gene set we determine a 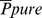 using the procedure described above. The *P_random_* is the proportion of 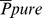 at least as significant as the true observed *P_pure_* for the candidate gene set (**Figure 1B**).
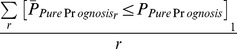
Third, we compute the *P_enrichment_* to indicate if a candidate gene set is enriched for prognostic genes. While the procedure to compute the *P_pure_* uses the label determined by k-means clustering with a candidate gene set as a binary feature to correlate with survival, the procedure to compute the *P_enrichment_* uses the univariate prognostic association of genes within a candidate gene to produce a gene set enrichment score to indicate the degree to which a gene set is enriched for genes that show strong univariate associations with survival (**Figure 1C**). To compute the *P_enrichment_*, we first rank all the genes in our meta-dataset according to their concordance index by using the function concordance.index in the *survcomp* package in R [16]. The concordance index of a gene represents the probability that, for a pair of patients randomly selected in our dataset, the patient whose tumor expresses that gene at a higher level will experience the appearance of distant metastasis or death before the other patient. Based on this genome-wide ranking we perform a pre-ranked GSEA [17], [18] to identify the candidate gene sets that are significantly enriched in genes with either significantly low or high concordance indices. The GSEA procedure for SAPS has two basic steps. First, an enrichment score is computed to indicate the overrepresentation of a candidate gene set at the top or bottom extremes of the ranked list of concordance indices. This enrichment score is normalized to account for a candidate gene set's size. Second, the statistical significance of the normalized enrichment score is estimated by permuting the genes to generate the *P_enrichment_* (see Refs. [17], [18] for further description of pre-ranked GSEA procedure), which indicates the probability that a similarly sized random gene set would achieve at least as extreme a normalized enrichment score as the candidate gene set (**Figure 1C**).

The *SAPS_score_* for each candidate gene set is then computed as the negative log_10_ of the maximum of the (*P_pure_*, *P_random_*, and *P_enrichment_*) times the direction of the association (positive or negative) (**Figure 1D**). For a given candidate gene set, the *SAPS_score_* specifies the direction of the prognostic association as well as indicates the raw *P-Value* achieved on all 3 of the (*P_pure prognosis_*, *P_random prognosis_*, and *P_enrichment_*). Since we take the negative log_10_ of the maximum of the (*P_pure prognosis_*, *P_random prognosis_*, and *P_enrichment_*), the larger the absolute value of the *SAPS_score_* the more significant the prognostic association of all 3 *P-Values*. The statistical significance of the *SAPS_score_* is determined by permuting genes, generating a null distribution for the *SAPS_score_* and computing the proportion of similarly sized gene sets from the null distribution achieving at least as large an absolute value of the *SAPS_score_* as that observed with the candidate gene set. When multiple candidate gene sets are evaluated, after generating each gene set's raw *SAPS_P-Value_* by permutation testing, we account for multiple hypotheses and control the false discovery rate using the method of Benjamini and Hochberg [19] to generate the *SAPS_q-value_* (**Figure 1E**). In our experiments, we have required a minimum absolute value (SAPS_score_) of greater than 1.3 and a maximum *SAPS_q-value_* of less than 0.05 to consider a gene set prognostically significant. These thresholds ensure that a significant prognostic gene set will have achieved a raw *P-Value* of less than or equal to 0.05 for each of *P_pure_*, *P_random_*, and *P_enrichment_*, and will have achieved an overall *SAPS_q-Value_* of less than or equal to 0.05.

### Application and Validation

We chose two model systems to investigate the performance of SAPS. The first is a curated sample of breast cancer datasets previously described in Haibe-Kains *et al.* [20]. Our analysis focused on nineteen datasets with patient survival information (total n = 3832) (**Table S1**). The second dataset was a compendium of twelve ovarian cancer datasets with survival data, as described in Bentink et al. [21], which includes data from 1735 ovarian cancer patients for whom overall survival data were available (**Table S2**).

### Identifying Molecular Subtypes

In breast cancer, we used SCMGENE [20] as implemented in the R/Bioconductor *genefu* package [22] to assign patients to one of four molecular subtypes: ER+/HER2− low proliferation, ER+/HER2− high proliferation, ER−/HER2− and HER2+. In ovarian cancer, we used the ovcAngiogenic model [21] as implemented in *genefu* to classify patients as having disease of either angiogenic or non-angiogenic subtype.

### Data Scaling and Merging

One challenge in the analysis of large published datasets is the heterogeneity of the platforms used to collect data (see **Table S1 and Table S2**). To standardize the data, we used normalized log_2_(intensity) for single-channel platforms and log_2_(ratio) in dual-channel platforms. Hybridization probes were mapped to Entrez GeneID as described in Shi et al. [23] using RefSeq and Entrez whenever possible; otherwise mapping was performed using IDconverter (http://idconverter.bioinfo.cnio.es) [24]. When multiple probes mapped to the same Entrez GeneID, we used the one with the highest variance in the dataset under study.

To allow for simultaneous analysis of datasets from multiple institutions, we tested two data merging protocols. First, we scaled and centered each expression feature across all patients in each dataset (standard *Z* scores), and we merged the scaled data from the different datasets (“traditional scaling”). In a second scaling procedure, we first assigned each patient in each data set to a breast or ovarian cancer molecular subtype, using the SCMGENE [20] and ovcAngiogenic [21] models, respectively. We then scaled and centered each expression feature separately within a specific molecular subtype within each dataset, so that each expression value was transformed into a Z score indicating the level of expression within patients of a specific molecular subtype within a dataset (“subtype-specific scaling”).

After merging datasets, we removed genes with missing data in more than half of the samples and we removed samples that were missing data on more than half of the genes or for which there was no information on distant metastasis free survival (for breast) or overall survival (for ovarian). The resulting breast cancer dataset contained 2731 cases with 13091 unique Entrez gene IDs and the ovarian cancer dataset had 1670 cases and 11247 unique Entrez gene IDs for. For each of these reduced data matrices, we estimated missing values using the function knn.impute in the impute package in R [25].

Given that breast cancer is an extremely heterogeneous disease with well-defined disease subtypes, and a primary objective of our work is to identify subtype-specific prognostic pathways in breast cancer, we focus our subsequent analyses on the subtype-specific scaled data. Given that ovarian cancer subtypes are more subtle and less well defined than breast cancer molecular subtypes, we focus our subsequent analyses in ovarian cancer on the traditional scaled data. SAPS scores in breast and ovarian cancer generated from the two different scaling procedures showed moderate to strong correlation across the breast and ovarian cancer molecular subtypes.

### Gene Sets

We downloaded gene sets from the Molecular Signatures Database (MSigDB) [17] (http://www.broadinstitute.org/gsea/msigdb/collections.jsp) (“molsigdb.v3.0.entrez.gmt”). MSigDB contains 5 major collections (positional gene sets, curated gene sets, motif gene sets, computational gene sets, and GO gene sets) comprising of a total of 6769 gene sets. We limited our analysis to gene sets with less than or equal to 250 genes and valid data for genes included in the meta-data sets, resulting in 5320 gene sets in the breast cancer analysis and 5355 in the ovarian cancer analysis.

### Application of SAPS to Breast Cancer

**Figure 2:**
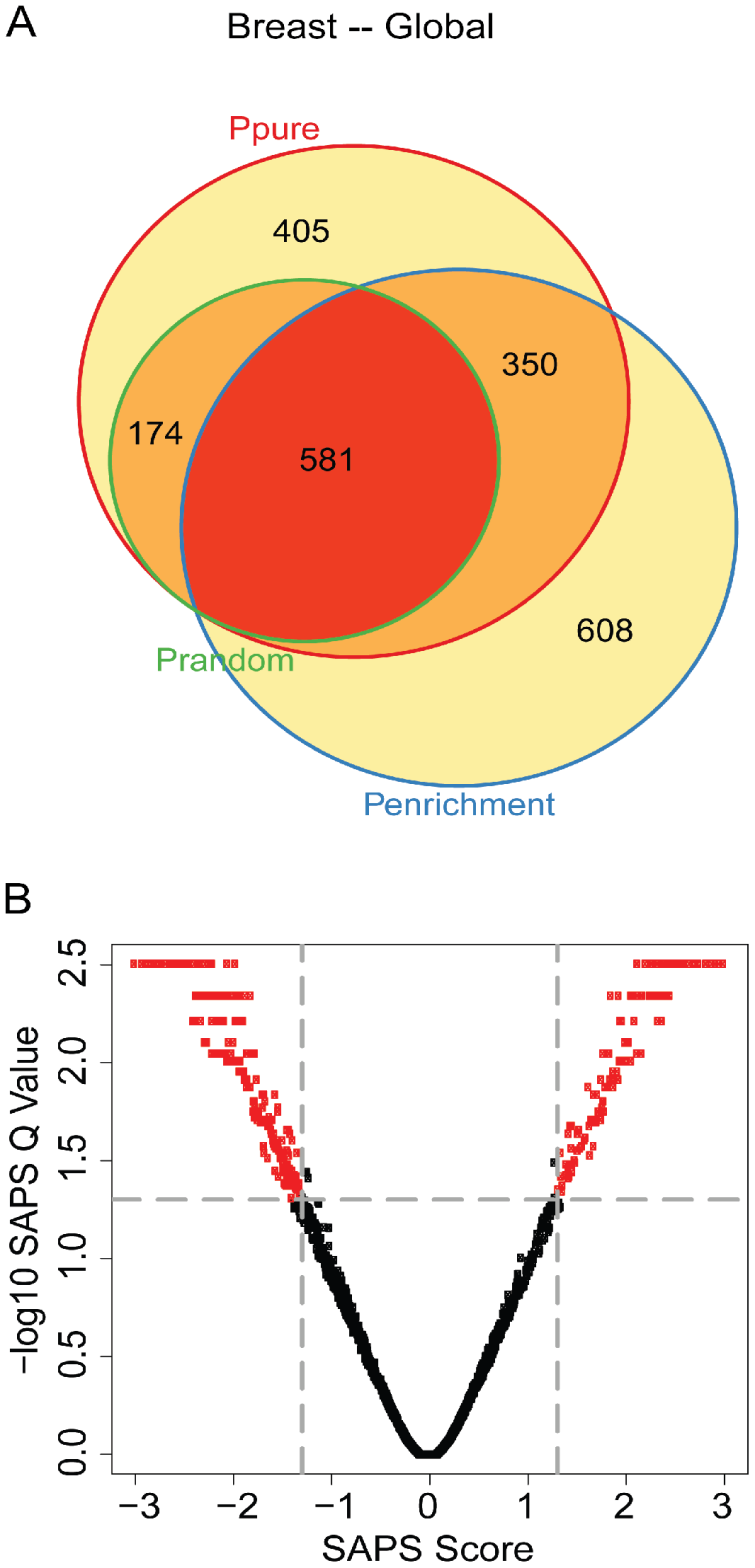
Global breast cancer Venn diagram and scatterplot. (**A**) The gene sets significant by at least one of the P values at the 0.05 level are displayed in a Venn diagram. (**B**) The −log10 of the *SAPS_q-value_* is plotted on the y-axis and the *SAPS_score_* along the x axis for each of the 5320 gene sets in the Molecular Signatures Database for their prognostic significance in breast cancer overall. Each point in the scatterplot represents a gene set, and gene sets that achieved a *SAPS_q-value_*≤0.05 and an absolute value (*SAPS_score_*)≥1.3 are colored in red.

We first applied SAPS to the entire collection of breast cancer cases independent of subtype. Of the 5320 gene sets evaluated, 1510 (28%) achieved a raw *P-Value* of 0.05 by *P_pure_*, 1539 (29%) by *P_enrichment_*, 755 (14%) by *P_random_*, 581 (11%) by all 3 raw *P-Values*, and 564 (11%) of these are significant at the *SAPS_q-value_* of 0.05 **(** **Figure 2** **)**.

The top-ranked gene sets identified by SAPS and associated with poor prognosis in all breast cancers independent of subtype contained gene sets previously found to be associated with poor prognosis in breast cancer (**Table 1**). Thus it is not surprising that these emerged as the most significant, and this result serves as a measure of validation. We note that the list of top gene sets associated with poor breast cancer prognosis identified in our overall analysis includes the gene set VANTVEER_BREAST_CANCER_METASTASIS_DN, which according to the Molecular Signatures Database website is defined as “Genes whose expression is significantly and negatively correlated with poor breast cancer clinical outcome (defined as developing distant metastases in less than 5 years).” Our analysis suggests that the set of genes is positively correlated with poor breast cancer clinical outcome. Comparison the gene list to the published “poor prognosis” gene list from van't Veer et al. [26] confirms that the gene list is mislabeled in the Molecular Signatures Database and is in fact the set of genes positively associated with metastasis in van't Veer *et al.* [26]

**Table 1:** Top prognostic signatures in global breast cancer.

	Size	*SAPS_q-value_*	*SAPS_score_*	*P_pure_*	*P_random_*	*P_enrichment_*	Dir
NADERI_BREAST_CANCER_PROGNOSIS_UP	34	0.0031	3	2.7E-09	0.0001	0.001	Poor
HAHTOLA_MYCOSIS_FUNGOIDES_DN	16	0.0031	−3	4.4E-09	0.0001	0.001	Good
VANTVEER_BREAST_CANCER_POOR_PROGNOSIS	44	0.0031	3	1.1E-08	0.0001	0.001	Poor
LU_TUMOR_VASCULATURE_DN	9	0.0031	−3	2.8E-08	0.0002	0.001	Good
MILICIC_FAMILIAL_ADENOMATOUS_POLYPOSIS_DN	9	0.0031	−3	4.3E-08	0.0003	0.001	Good
VANTVEER_BREAST_CANCER_METASTASIS_DN	100	0.0031	3	5E-08	0.0001	0.001	Poor
SEMBA_FHIT_TARGETS_DN	9	0.0031	3	7.5E-08	0.0003	0.001	Poor
BIOCARTA_IL2RB_PATHWAY	38	0.0031	−3	8.6E-08	0.0001	0.001	Good
SOTIRIOU_BREAST_CANCER_GRADE_1_VS_3_UP	150	0.0031	3	1E-07	0.0001	0.001	Poor
CELL_DIVISION	19	0.0031	3	1.3E-07	0.0008	0.001	Poor

Gene sets in the analysis come from the Broad institute's MSigDB. These gene sets can be further evaluated at http://www.broadinstitute.org/gsea/msigdb/search.jsp.

The top-ranking gene sets associated with good prognosis were not originally identified in breast cancers, and represent a range of biological processes. Several were from analyses of hematolymphoid cells, including: genes down-regulated in monocytes isolated from peripheral blood samples of patients with mycosis fungoides compared to those from normal healthy donors, genes associated with the IL-2 receptor beta chain in T cell activation, and genes down-regulated in B2264-19/3 cells (primary B lymphocytes) within 60–180 min after activation of LMP1 (an oncogene encoded by Epstein Barr virus). These gene sets suggest that specific subsets of immune system activation are associated with improved breast cancer prognosis, consistent with reports that the presence infiltrating lymphocytes is predictive of outcome in many cancers.

**Figure 3:**
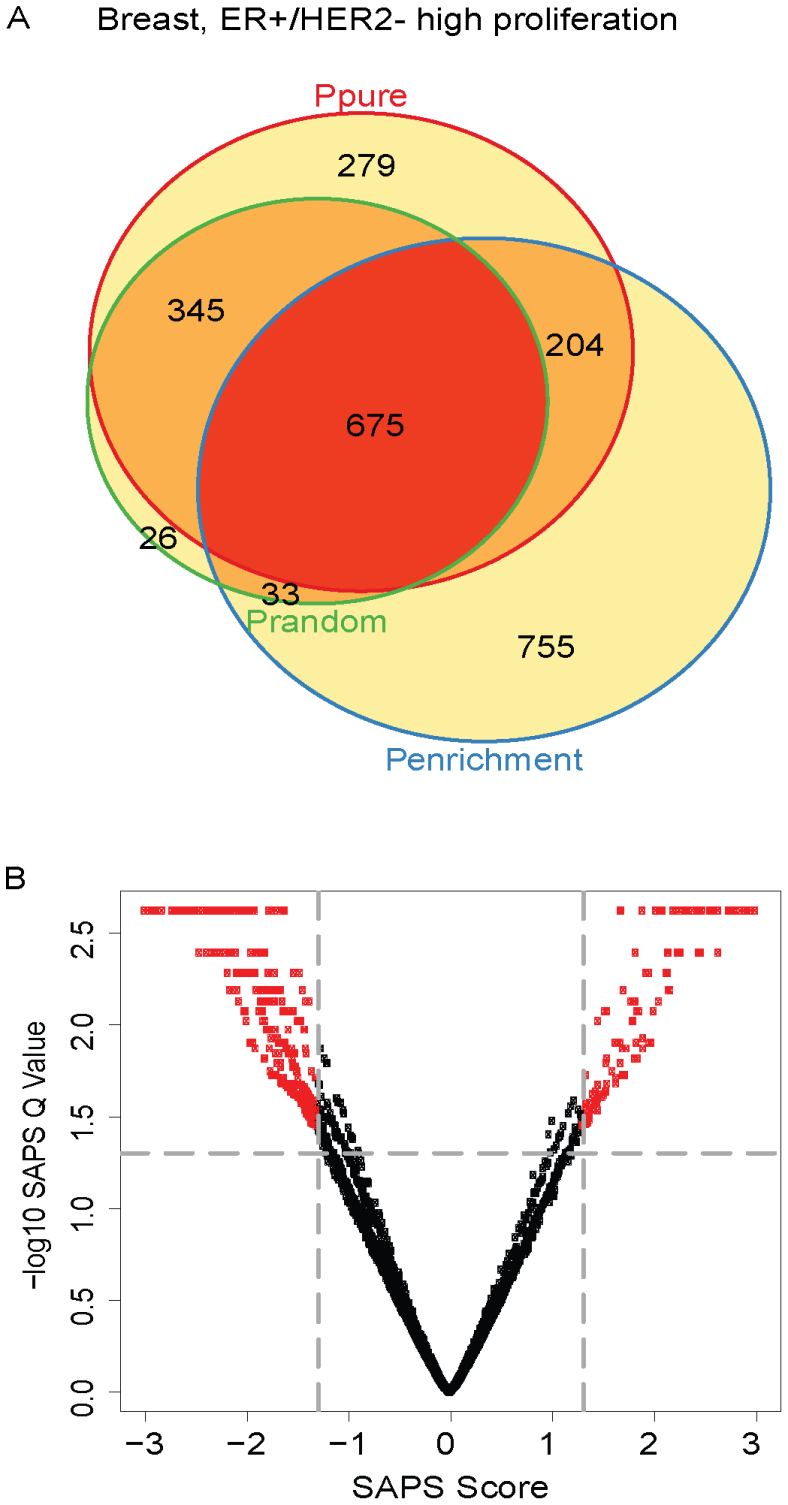
ER+/HER2− high proliferation Venn diagram and scatterplot. (**A**) The gene sets significant by at least one of the P values at the 0.05 level are displayed in a Venn diagram. (**B**) The −log10 of the *SAPS_q-value_* is plotted on the y-axis and the *SAPS_score_* along the x axis for each of the 5320 gene sets in the Molecular Signatures Database for their prognostic significance in the ER+/HER2− breast cancer molecular subtype. Each point in the scatterplot represents a gene set, and gene sets that achieved a *SAPS_q-value_*≤0.05 and an absolute value (*SAPS_score_*)≥1.3 are colored in red.

We then applied SAPS to the ER+/HER2− high proliferation subtype. Of the 5320 gene sets evaluated, 1503 (28%) achieved a raw *P-Value* of 0.05 by *P_pure_*, 1667 (31%) by *P_enrichment_*, 1079 (20%) by *P_random_*, 675 (13%) by all 3 raw *P-Values*, and all 675 of these are significant at the *SAPS_q-value_* of 0.05. The top-ranking gene sets by SAPS*_score_* are associated with cancer and proliferation. One of the top-ranking gene sets was associated with Ki67, a well-known prognostic marker in Luminal B breast cancers [27]. Overall, the patterns of significance are highly similar to that seen in breast cancer analyzed independent of subtype (**Figure 3** **,** **Table 2**).

**Table 2:** Top prognostic signatures in ER+/HER2− high proliferation.

	Size	*SAPS_q-value_*	*SAPS_score_*	*P_pure_*	*P_random_*	*P_enrichment_*	Dir
LOPEZ_MESOTELIOMA_SURVIVAL_TIME_UP	14	0.0024	3	9.3E-10	0.0001	0.001	Poor
VANTVEER_BREAST_CANCER_POOR_PROGNOSIS	44	0.0024	3	5.5E-09	0.0001	0.001	Poor
MONTERO_THYROID_CANCER_POOR_SURVIVAL_UP	9	0.0024	3	1.1E-08	0.0001	0.001	Poor
VANTVEER_BREAST_CANCER_METASTASIS_DN	100	0.0024	3	3.3E-08	0.0001	0.001	Poor
GNF2_MKI67	25	0.0024	3	6.6E-08	0.0001	0.001	Poor
NADERI_BREAST_CANCER_PROGNOSIS_UP	34	0.0024	3	8.3E-08	0.0001	0.001	Poor
CHANG_CYCLING_GENES	38	0.0024	3	1.3E-07	0.0001	0.001	Poor
LY_AGING_MIDDLE_DN	15	0.0024	3	1.6E-07	0.0001	0.001	Poor
GNF2_CENPE	36	0.0024	3	1.7E-07	0.0001	0.001	Poor
CHEMNITZ_RESPONSE_TO_PROSTAGLANDIN_E2_UP	120	0.0024	3	2E-07	0.0001	0.001	Poor

Gene sets in the analysis come from the Broad institute's MSigDB. These gene sets can be further evaluated at http://www.broadinstitute.org/gsea/msigdb/search.jsp.

**Figure 4:**
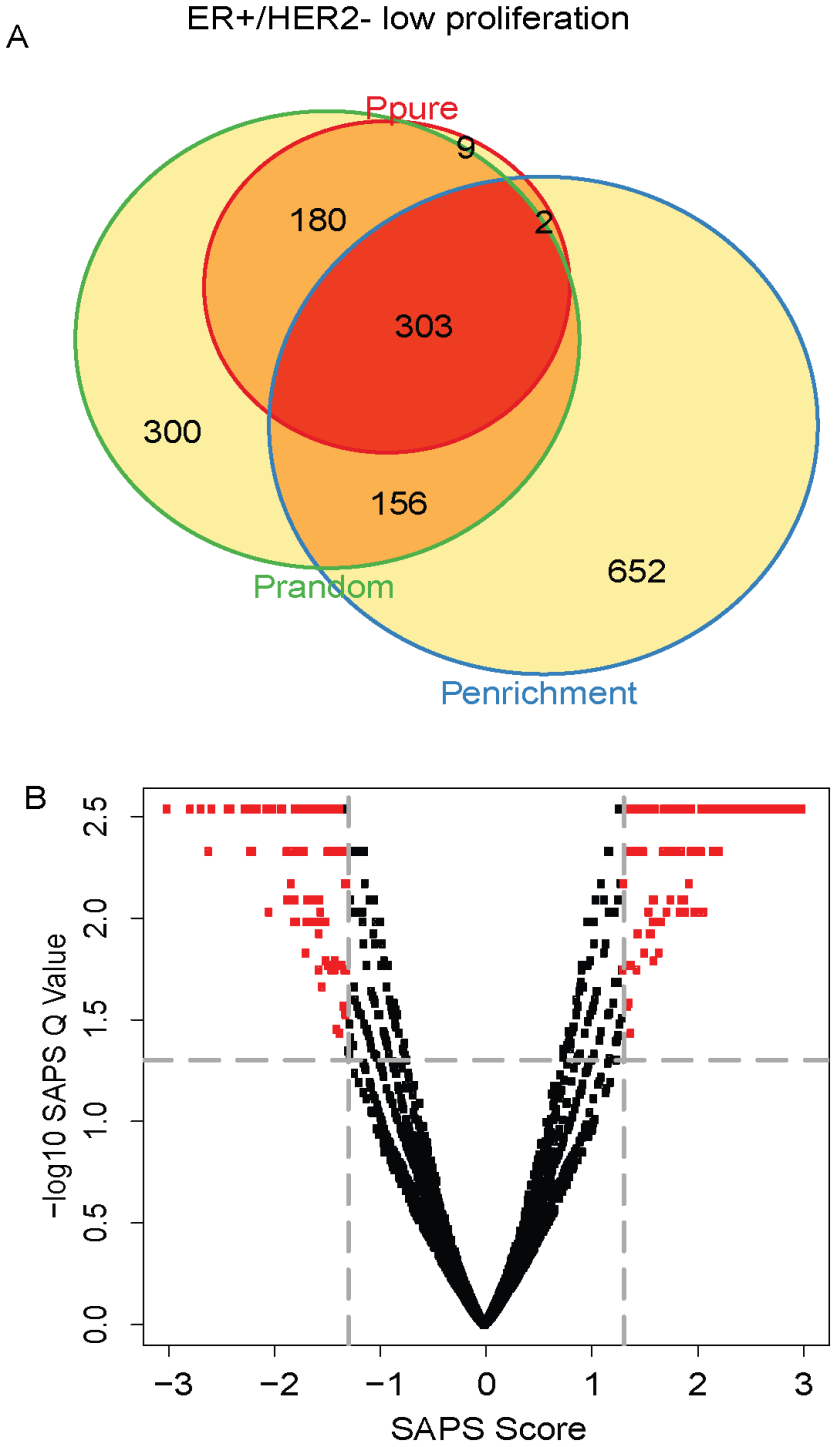
ER+/HER2− low proliferation Venn diagram and scatterplot. (**A**) The gene sets significant by at least one of the P values at the 0.05 level are displayed in a Venn diagram. (**B**) The −log10 of the *SAPS_q-value_* is plotted on the y-axis and the *SAPS_score_* along the x axis for each of the 5320 gene sets in the Molecular Signatures Database for their prognostic significance in the ER+/HER2− low proliferation breast cancer molecular subtype. Each point in the scatterplot represents a gene set, and gene sets that achieved a *SAPS_q-value_*≤0.05 and an absolute value (*SAPS_score_*)≥1.3 are colored in red.

Next, we used SAPS to analyze the ER+/HER2− low proliferation samples. Of the 5320 gene sets evaluated, 494 (9%) achieved a raw *P-Value* of 0.05 by *P_pure_*, 1113 (21%) by *P_enrichment_*, 939 (18%) by *P_random_*, 303 (6%) by all 3 raw *P-Values*, and all 303 of these were significant at the *SAPS_q-value_* of 0.05. The top-ranking ER+/HER2− low proliferation prognostic gene sets by SAPSscore are also highly enriched for genes involved in proliferation (**Figure 4** **,** **Table 3**). Top ranking gene sets associated with good prognosis include those highly expressed in lobular breast carcinoma relative to ductal and inflammation-associated genes up-regulated following infection with human cytomegalovirus.

**Table 3:** Top prognostic signatures in ER+/HER2 low proliferation.

	Size	*SAPS_q-value_*	*SAPS_score_*	*P_pure_*	*P_random_*	*P_enrichment_*	Dir
BERTUCCI_INVASIVE_CARCINOMA_DUCTAL_VS_LOBULAR_DN	43	0.0029	−3	0.000029	0.0001	0.001	Good
MITOTIC_SPINDLE_ORGANIZATION_AND_BIOGENESIS	8	0.0029	3	0.000041	0.0001	0.001	Poor
KINESIN_COMPLEX	14	0.0029	3	0.000055	0.0001	0.001	Poor
M_PHASE	98	0.0029	3	0.000068	0.0001	0.001	Poor
MORF_BUB1B	61	0.0029	3	0.000085	0.0001	0.001	Poor
BENPORATH_PROLIFERATION	130	0.0029	3	0.00014	0.0001	0.001	Poor
BROWNE_HCMV_INFECTION_2HR_UP	39	0.0029	−3	0.00039	0.0001	0.001	Good
CHROMOSOME_SEGREGATION	28	0.0029	3	0.00036	0.0001	0.001	Poor
CHROMOSOMEPERICENTRIC_REGION	30	0.0029	3	0.00024	0.0001	0.001	Poor
FERREIRA_EWINGS_SARCOMA_UNSTABLE_VS_STABLE_UP	120	0.0029	3	0.00081	0.0001	0.001	Poor

Gene sets in the analysis come from the Broad institute's MSigDB. These gene sets can be further evaluated at http://www.broadinstitute.org/gsea/msigdb/search.jsp.

**Figure 5:**
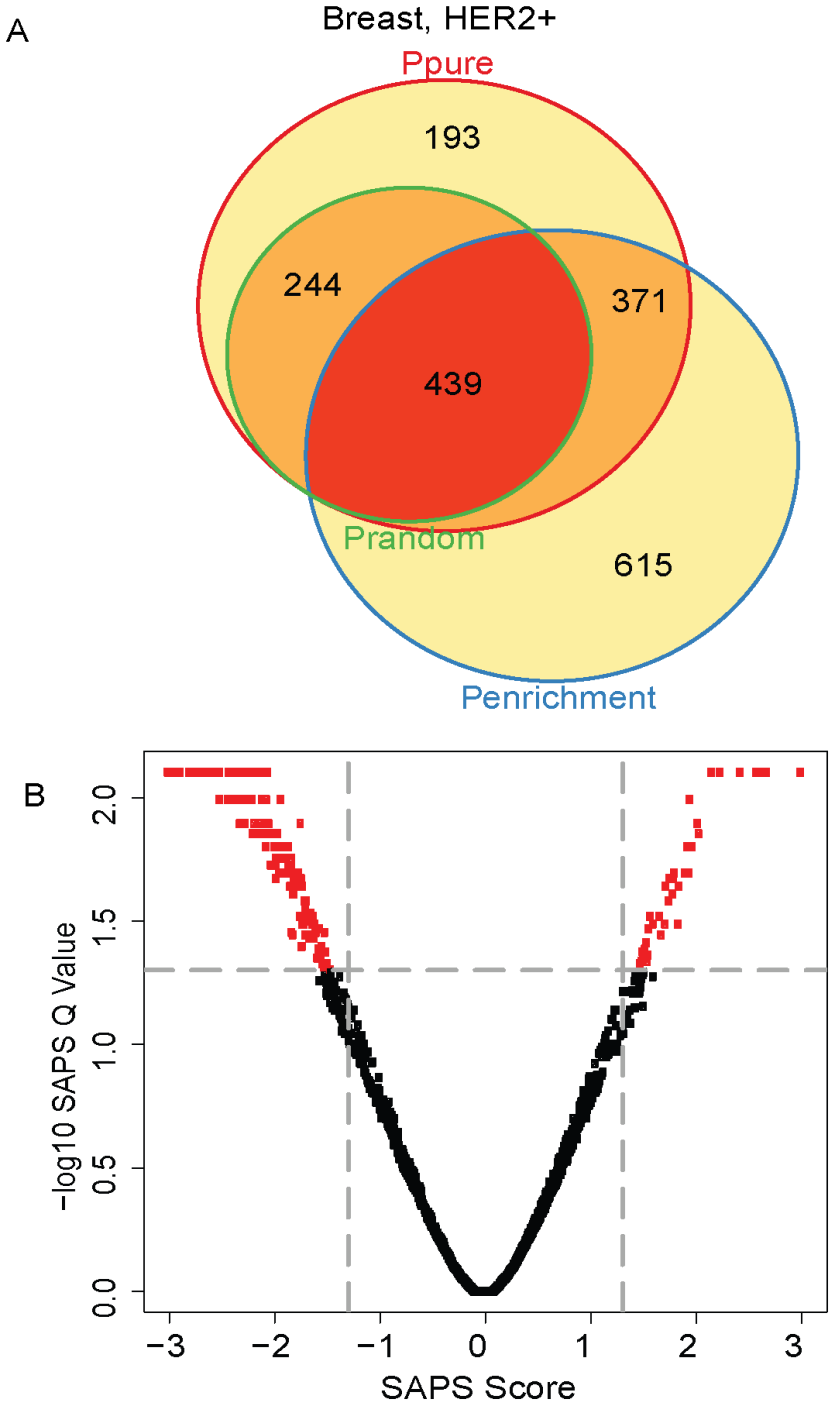
HER2+ Venn diagram and scatterplot. (**A**) The gene sets significant by at least one of the P values at the 0.05 level are displayed in a Venn diagram. (**B**) The −log10 of the *SAPS_q-value_* is plotted on the y-axis and the *SAPS_score_* along the x axis for each of the 5320 gene sets in the Molecular Signatures Database for their prognostic significance in the HER2+ breast cancer molecular subtype. Each point in the scatterplot represents a gene set, and gene sets that achieved a *SAPS_q-value_*≤0.05 and an absolute value (*SAPS_score_*)≥1.3 are colored in red.

Then, we applied SAPS to the HER2+ subset. Of the 5320 gene sets evaluated, 1247 (23%) achieved a raw *P-Value* of 0.05 by *P_pure_*, 1425 (27%) by *P_enrichment_*, 683 (13%) by *P_random_*, 439 (8%) by all 3 raw *P-Values*, and 342 (6%) of these are significant at the *SAPS_q-value_* of 0.05. Most of the top-ranking prognostic pathways in the HER2+ group by SAPS*_score_* are associated with better prognosis and include several gene sets associated with inflammatory response (**Figure 5** **,** **Table 4**). A gene set containing genes down-regulated in multiple myeloma cell lines treated with the hypomethylating agents decitabine and trichostatin A was significantly associated with improved prognosis in HER2+ breast cancer. The top-ranking gene set associated with decreased survival is a hypoxia-associated gene set. Hypoxia is a well-known prognostic factor in breast cancer [28], [29], and our analysis suggests it shows a very strong association with survival in the HER2+ breast cancer molecular subtype.

**Table 4:** Top prognostic signatures in HER2+.

	Size	*SAPS_q-value_*	*SAPS_score_*	*P_pure_*	*P_random_*	*P_enrichment_*	Dir
GNF2_ATM	26	0.0079	−3	2.4E-08	0.0001	0.001	Good
TSAI_RESPONSE_TO_IONIZING_RADIATION	120	0.0079	−3	3.1E-08	0.0001	0.001	Good
ZHU_CMV_ALL_UP	60	0.0079	−3	2.2E-07	0.0001	0.001	Good
WINTER_HYPOXIA_UP	75	0.0079	3	2.8E-07	0.0001	0.001	Poor
ZHANG_RESPONSE_TO_IKK_INHIBITOR_AND_TNF_UP	180	0.0079	−3	6E-07	0.0001	0.001	Good
GEISS_RESPONSE_TO_DSRNA_UP	30	0.0079	−3	7.9E-07	0.0001	0.001	Good
FARMER_BREAST_CANCER_CLUSTER_1	43	0.0079	−3	1.1E-06	0.0001	0.001	Good
HELLER_HDAC_TARGETS_SILENCED_BY_METHYLATION_DN	240	0.0079	−3	1.4E-06	0.0002	0.001	Good
JAK_STAT_CASCADE	26	0.0079	−3	1.4E-06	0.0002	0.001	Good
HELLER_HDAC_TARGETS_DN	240	0.0079	−3	1.5E-06	0.0002	0.001	Good

Gene sets in the analysis come from the Broad institute's MSigDB. These gene sets can be further evaluated at http://www.broadinstitute.org/gsea/msigdb/search.jsp.

**Figure 6:**
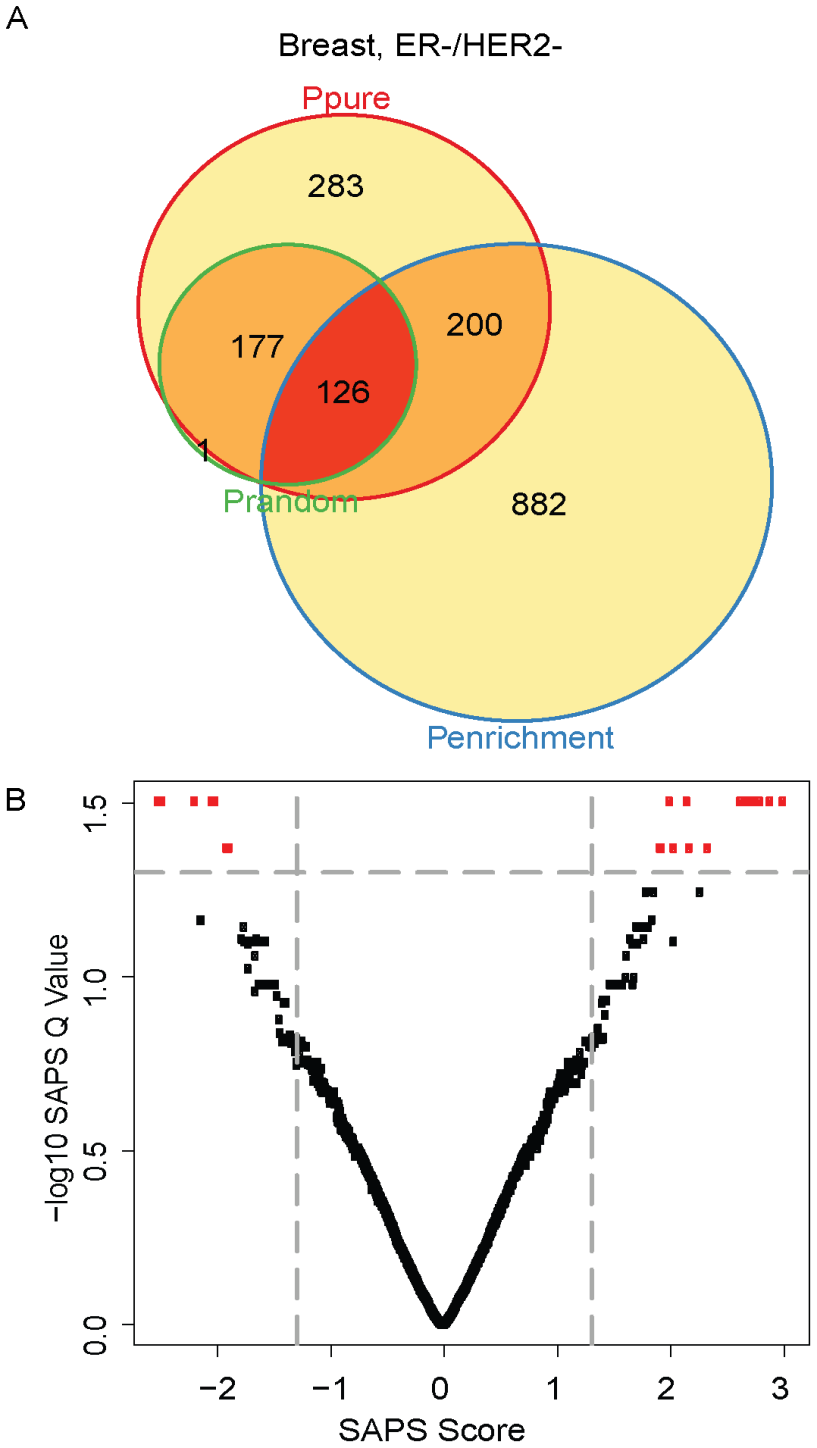
ER−/HER2− Venn diagram and scatterplot. (**A**) The gene sets significant by at least one of the P values at the 0.05 level are displayed in a Venn diagram. (**B**) The −log10 of the *SAPS_q-value_* is plotted on the y-axis and the *SAPS_score_* along the x axis for each of the 5320 gene sets in the Molecular Signatures Database for their prognostic significance in the ER−/HER2− breast cancer molecular subtype. Each point in the scatterplot represents a gene set, and gene sets that achieved a *SAPS_q-value_*≤0.05 and an absolute value (*SAPS_score_*)≥1.3 are colored in red.

Finally, we used SAPS to analyze the poor-prognosis “basal like” subtype which was classified as being ER−/HER2−. Of the 5320 gene sets evaluated, 786 (15%) achieved a raw *P-Value* of 0.05 by *P_pure_*, 1208 (23%) by *P_enrichment_*, 304 (6%) by *P_random_*, 126 (2%) by all 3 raw *P-Values*, and 25 (0.5%) of these are significant at the *SAPS_q-value_* of 0.05. Top-ranking gene sets associated with poor survival include genes up-regulated in MCF7 breast cancer cells treated with hypoxia mimetic DMOG, genes down-regulated in MCF7 cells after knockdown of HIF1A and HIF2A, genes regulated by hypoxia based on literature searches, genes up-regulated in response to both hypoxia and overexpression of an active form of HIF1A, and genes down-regulated in fibroblasts with defective XPC (an important DNA damage response protein) in response to cisplatin (**Figure 6** **,** **Table 5**). This analysis suggests that hypoxia-associated gene sets are key drivers of poor prognosis in HER2+ and ER−/HER2− breast cancer subtypes. Interestingly, cisplatin is an agent with activity in ER−/HER2− breast cancer, and it is has been suggested that ER−/HER2− breast cancers with defective DNA repair may show increased susceptibility to cisplatin [30].

**Table 5:** Top prognostic signatures in ER−/HER2−.

	Size	*SAPS_q-value_*	*SAPS_score_*	*P_pure_*	*P_random_*	*P_enrichment_*	Dir
ELVIDGE_HYPOXIA_BY_DMOG_UP	130	0.031	3	0.000089	0.0003	0.001	Poor
WANG_CISPLATIN_RESPONSE_AND_XPC_DN	140	0.031	3	0.00009	0.0003	0.001	Poor
ELVIDGE_HIF1A_AND_HIF2A_TARGETS_DN	100	0.031	3	0.00024	0.0008	0.001	Poor
WINTER_HYPOXIA_METAGENE	210	0.031	3	0.00064	0.0003	0.001	Poor
ELVIDGE_HIF1A_TARGETS_DN	87	0.031	2.9	0.00014	0.0013	0.001	Poor
GCM_MLL	110	0.031	2.8	0.00072	0.0016	0.001	Poor
GTATTAT,MIR-369-3P	140	0.031	2.7	0.00082	0.0018	0.001	Poor
CHEN_HOXA5_TARGETS_9HR_UP	220	0.031	2.7	0.0021	0.0021	0.001	Poor
MANALO_HYPOXIA_UP	200	0.031	2.6	0.0024	0.0021	0.001	Poor
CHAUHAN_RESPONSE_TO_METHOXYESTRADIOL_DN	96	0.031	−2.5	0.00057	0.003	0.0031	Good

Gene sets in the analysis come from the Broad institute's MSigDB. These gene sets can be further evaluated at http://www.broadinstitute.org/gsea/msigdb/search.jsp.

### Application of SAPS to Ovarian Cancer

**Figure 7:**
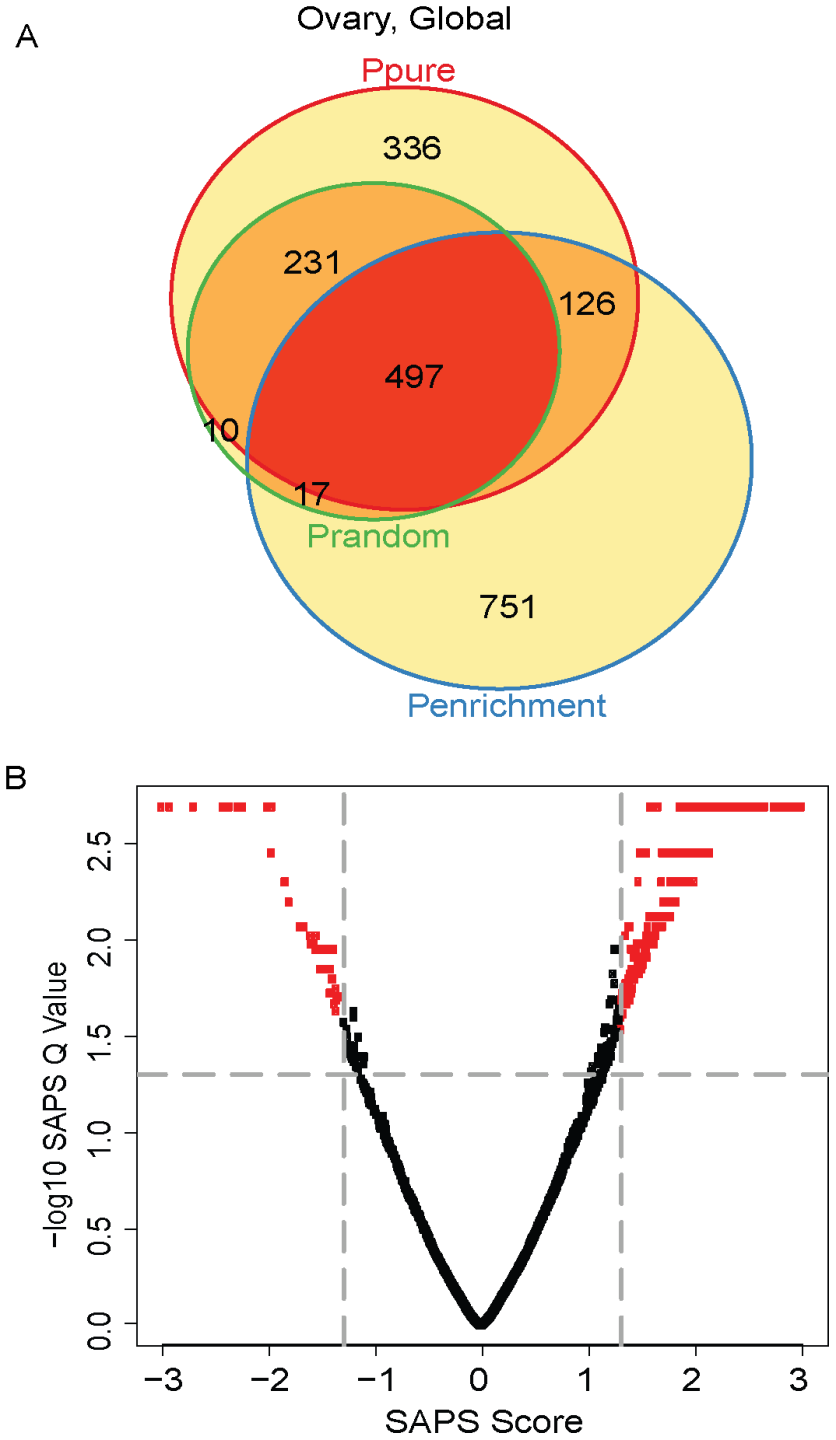
Global ovarian cancer Venn diagram and scatterplot. (**A**) The gene sets significant by at least one of the P values at the 0.05 level are displayed in a Venn diagram. (**B**) The −log10 of the *SAPS_q-value_* is plotted on the y-axis and the *SAPS_score_* along the x axis for each of the 5320 gene sets in the Molecular Signatures Database for their prognostic significance in ovarian cancer overall. Each point in the scatterplot represents a gene set, and gene sets that achieved a *SAPS_q-value_*≤0.05 and an absolute value (*SAPS_score_*)≥1.3 are colored in red.

Our analysis for ovarian cancer was similar to that for breast cancer. We began by applying SAPS to the entire collection of ovarian cancer samples independent of subtype. Of the 5355 gene sets evaluated, 1190 (22%) achieved a raw *P-Value* of 0.05 by *P_pure_*, 1391 (26%) by *P_enrichment_*, 755 (14%) by *P_random_*, 497 (9%) by all 3 raw *P-Values* (**Figure 7** **,** **Table 6**), and all 497 of these are significant at the *SAPS_q-value_* of 0.05. The top gene sets are involved in stem cell-related pathways and pathways related to epithelial-mesenchymal transition, including genes up-regulated in HMLE cells (immortalized non-transformed mammary epithelium) after E-cadhedrin (CDH1) knockdown by RNAi, genes down-regulated in adipose tissue mesenchymal stem cells vs. bone marrow mesenchymal stem cells, genes down-regulated in medullary breast cancer relative to ductal breast cancer, genes down-regulated in basal-like breast cancer cell lines as compared to the mesenchymal-like cell lines, genes up-regulated in metaplastic carcinoma of the breast subclass 2 compared to the medullary carcinoma subclass 1, and genes down-regulated in invasive ductal carcinoma compared to invasive lobular carcinoma.

**Table 6:** Top prognostic signatures in global ovarian cancer.

	Size	*SAPS_q-value_*	*SAPS_score_*	*P_pure_*	*P_random_*	*P_enrichment_*	Dir
V$HOX13_01	25	0.002	3	7.4E-11	0.0001	0.001	Poor
VECCHI_GASTRIC_CANCER_ADVANCED_VS_EARLY_UP	120	0.002	3	1.3E-10	0.0001	0.001	Poor
ONDER_CDH1_TARGETS_2_UP	230	0.002	3	3.1E-10	0.0001	0.001	Poor
IZADPANAH_STEM_CELL_ADIPOSE_VS_BONE_DN	84	0.002	3	3.1E-10	0.0001	0.001	Poor
BERTUCCI_MEDULLARY_VS_DUCTAL_BREAST_CANCER_DN	120	0.002	3	4.5E-10	0.0001	0.001	Poor
BROWNE_HCMV_INFECTION_24HR_DN	140	0.002	3	1.2E-09	0.0001	0.001	Poor
CHARAFE_BREAST_CANCER_BASAL_VS_MESENCHYMAL_DN	39	0.002	3	1.2E-09	0.0001	0.001	Poor
LIEN_BREAST_CARCINOMA_METAPLASTIC	29	0.002	3	2.4E-09	0.0001	0.001	Poor
SENESE_HDAC1_AND_HDAC2_TARGETS_DN	170	0.002	3	3.6E-09	0.0001	0.001	Poor
BERTUCCI_INVASIVE_CARCINOMA_DUCTAL_VS_LOBULAR_DN	40	0.002	3	4.6E-09	0.0001	0.001	Poor

Gene sets in the analysis come from the Broad institute's MSigDB. These gene sets can be further evaluated at http://www.broadinstitute.org/gsea/msigdb/search.jsp.

**Figure 8:**
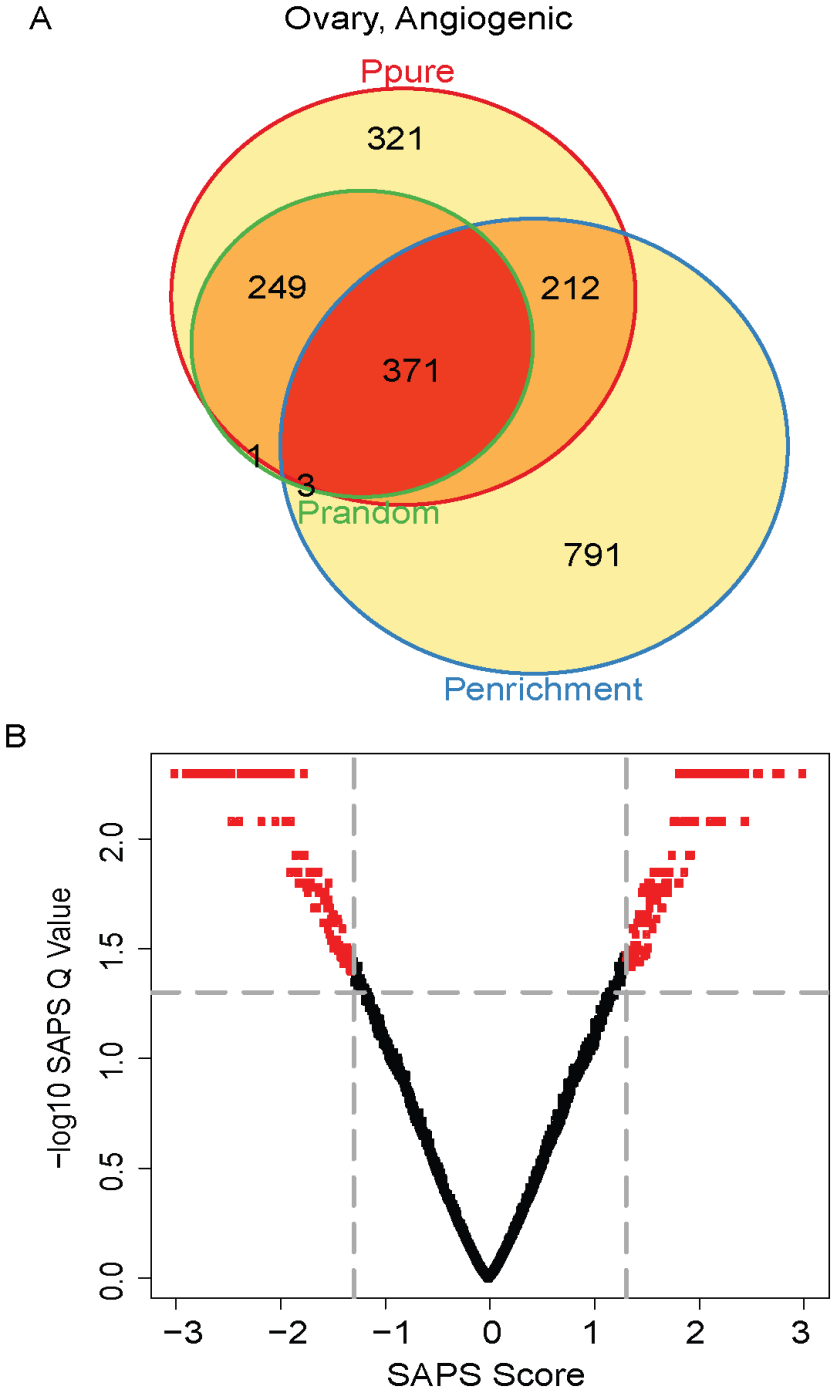
Angiogenic subtype Venn diagram and scatterplot. (**A**) The gene sets significant by at least one of the P values at the 0.05 level are displayed in a Venn diagram. (**B**) The −log10 of the *SAPS_q-value_* is plotted on the y-axis and the *SAPS_score_* along the x axis for each of the 5355 gene sets in the Molecular Signatures Database for their prognostic significance in the Angiogenic ovarian cancer molecular subtype. Each point in the scatterplot represents a gene set, and gene sets that achieved a *SAPS_q-value_*≤0.05 and an absolute value (*SAPS_score_*)≥1.3 are colored in red.

We then analyzed the angiogenic subtype. Of the 5355 gene sets evaluated, 1153 (22%) achieved a raw *P-Value* of 0.05 by *P_pure_*, 1377 (26%) by *P_enrichment_*, 624 (12%) by *P_random_*, 371 (7%) by all 3 raw *P-Values* (**Figure 7** **,** **Table 6**), and all of these are significant at the *SAPS_q-value_* of 0.05. Top-ranking gene sets associated with poor prognosis in the angiogenic subtype include: a set of targets of miR-33 (associated with poor prognosis) (**Figure 8** **,** **Table 7**). This microRNA has not previously been implicated in ovarian carcinogenesis. Other top hits include several immune response gene sets, which were associated with improved prognosis.

**Table 7:** Top prognostic signatures in Angiogenic overall.

	Size	*SAPS_q-value_*	*SAPS_score_*	*P_pure_*	*P_random_*	*P_enrichment_*	Dir
CAATGCA,MIR-33	68	0.0051	3	0.000012	0.0001	0.001	Poor
BIOCARTA_CTL_PATHWAY	13	0.0051	−3	0.000024	0.0005	0.001	Good
BIOCARTA_NO2IL12_PATHWAY	16	0.0051	−3	0.000041	0.0005	0.001	Good
BIOCARTA_IL12_PATHWAY	21	0.0051	−3	0.000042	0.0007	0.001	Good
HOSHIDA_LIVER_CANCER_SUBCLASS_S3	250	0.0051	−3	0.00007	0.0001	0.001	Good
FARMER_BREAST_CANCER_CLUSTER_1	36	0.0051	−3	0.000086	0.0006	0.001	Good
STTTCRNTTT_V$IRF_Q6	130	0.0051	−3	0.00023	0.0001	0.001	Good
FURUKAWA_DUSP6_TARGETS_PCI35_UP	50	0.0051	−3	0.00012	0.0005	0.001	Good
GNF2_RTN1	45	0.0051	3	0.00015	0.0008	0.001	Poor
ZHU_CMV_ALL_UP	53	0.0051	−3	0.00016	0.0007	0.001	Good

Gene sets in the analysis come from the Broad institute's MSigDB. These gene sets can be further evaluated at http://www.broadinstitute.org/gsea/msigdb/search.jsp.

**Figure 9:**
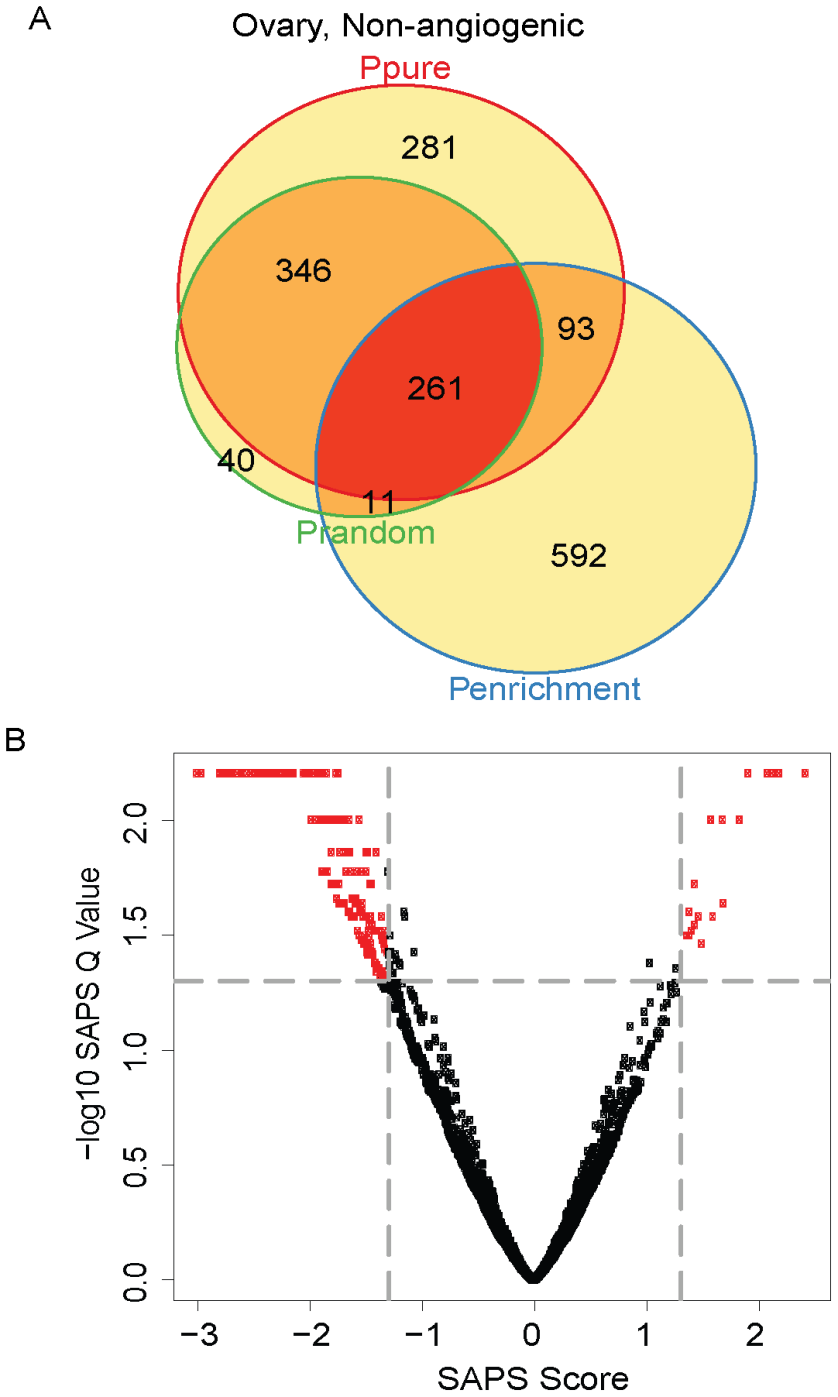
Non-angiogenic subtype Venn diagram and scatterplot. (**A**) The gene sets significant by at least one of the P values at the 0.05 level are displayed in a Venn diagram. (**B**) The −log10 of the *SAPS_q-value_* is plotted on the y-axis and the *SAPS_score_* along the x axis for each of the 535 gene sets in the Molecular Signatures Database for their prognostic significance in the Non-angiogenic ovarian cancer molecular subtype. Each point in the scatterplot represents a gene set, and gene sets that achieved a *SAPS_q-value_*≤0.05 and an absolute value (*SAPS_score_*)≥1.3 are colored in red.

Finally, we analyzed the non-angiogenic subtype of ovarian cancer. Of the 5355 gene sets evaluated, 981 (18%) achieved a raw *P-Value* of 0.05 by *P_pure_*, 957 (18%) by *P_enrichment_*, 658 (12%) by *P_random_*, 261 (5%) by all 3 raw *P-Values* (**Figure 7** **,** **Table 6**), and of these, 254 (5%) are significant at the *SAPS_q-value_* of 0.05 (**Figure 9** **,** **Table 8**). The top ranked pathways associated with improved survival are immune-related gene sets and a gene set found to be negatively associated with metastasis in head and neck cancers.

**Table 8:** Top prognostic signatures in Non-angiogenic overall.

	Size	*SAPS_q-value_*	*SAPS_score_*	*P_pure_*	*P_random_*	*P_enrichment_*	Direction
KEGG_ASTHMA	27	0.0062	−3	6.5E-07	0.0001	0.001	Good
BUDHU_LIVER_CANCER_METASTASIS_UP	8	0.0062	−3	7.3E-07	0.0001	0.001	Good
DEPHOSPHORYLATION	64	0.0062	−3	0.000015	0.0006	0.001	Good
ODONNELL_TARGETS_OF_MYC_AND_TFRC_UP	62	0.0062	−3	0.000021	0.0007	0.001	Good
HUPER_BREAST_BASAL_VS_LUMINAL_DN	55	0.0062	−3	0.000021	0.0007	0.001	Good
SENGUPTA_NASOPHARYNGEAL_CARCINOMA_DN	180	0.0062	−3	0.00011	0.0006	0.001	Good
WAMUNYOKOLI_OVARIAN_CANCER_LMP_UP	180	0.0062	−3	0.00011	0.0006	0.001	Good
OKUMURA_INFLAMMATORY_RESPONSE_LPS	170	0.0062	−3	0.00014	0.0009	0.001	Good
RICKMAN_METASTASIS_DN	200	0.0062	−3	0.00019	0.0008	0.001	Good
MCLACHLAN_DENTAL_CARIES_DN	210	0.0062	−3	0.00022	0.0009	0.001	Good

Gene sets in the analysis come from the Broad institute's MSigDB. These gene sets can be further evaluated at http://www.broadinstitute.org/gsea/msigdb/search.jsp.

### Integrated Analysis of Breast and Ovarian Cancer Prognostic Pathways

**Figure 10:**
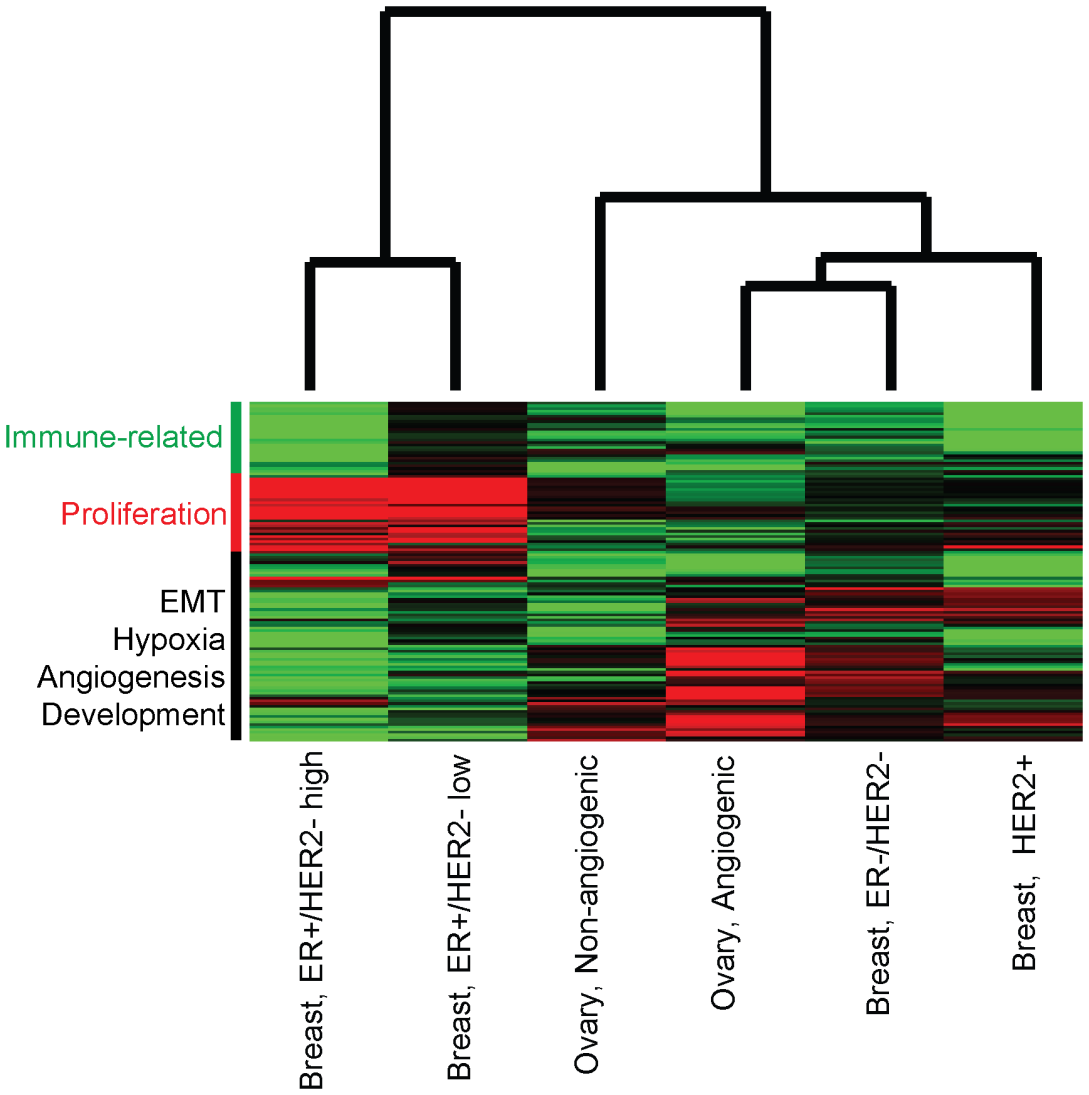
Hierarchical clustering of breast and ovarian cancers and their subtypes based on SAPS scores. Breast cancer and ovarian cancer molecular subtypes were clustered with the 1300 gene sets with absolute value (*SAPS_score_*)≥1.3 and *SAPS_q-value_*≤0.05 in at least one disease subtype. Hierarchical clustering was performed on the *SAPS_Score_*. In the heatmap, green indicates the gene set is associated with improved prognosis and red with poorer prognosis.

To assess similarities and differences in prognostic pathways in both breast and ovarian cancer molecular subtypes, we performed hierarchical clustering of the disease subtypes using *SAPS_scores_*. Specifically, we identified the 1300 gene sets with *SAPS_q-value_*≤0.05 and absolute value (*SAPS_score_*)≥1.3 in at least one of the breast and ovarian cancer molecular subtypes. We clustered the gene sets and disease subtypes using hierarchical clustering with complete linkage and distance defined as one minus Spearman rank correlation (**Figure 10**). This analysis shows two dominant clusters of disease subtypes, with one cluster containing ER+/HER2− high proliferation and ER+/HER2− low proliferation breast cancer molecular subtypes, and the second cluster containing ovarian cancer molecular subtypes and the ER−/HER2− and HER2+ breast cancer molecular subtypes. *SAPS_scores_* for within ER+ breast cancer molecular subtypes, within ER−/HER2− and HER2+ breast cancer molecular subtypes, and within ovarian cancer molecular subtypes show high correlation (Spearman rho = 0.61, 0.68, and 0.51, respectively, all p<2.2×10^−16^). Interestingly, the *SAPS_scores_* for the ER−/HER2− and HER2+ breast cancer subtypes show far greater correlation with the *SAPS_scores_* in the ovarian cancer molecular subtypes than with the *SAPS_scores_* in ER+ molecular subtypes (median Spearman rho is 0.5 for correlation of ER−/HER2− and HER2+ breast cancer molecular subtypes with ovarian cancer molecular subtypes vs. 0.16 for ER− molecular subtypes with ER+ molecular subtypes (**Figure 10**). This analysis demonstrates the importance of performing subtype-specific analyses in breast cancer, as breast cancer is an extremely heterogeneous disease and prognostic pathways in ER−/HER2− and HER2+ breast cancer subtypes are far more similar to prognostic pathways in ovarian cancer than with prognostic pathways in ER+ breast cancer subtypes. Recently, the TCGA breast cancer analysis demonstrated that the “basal” subtype of breast cancer (ER−/HER2−) showed genomic alterations far more similar to ovarian cancer than to other breast cancer molecular subtypes [31]. Our findings show that ER−/HER2− breast cancers share not only genomic alterations but also prognostic pathways with ovarian cancer.

Examining the clusters of gene sets with differential prognostic associations across breast and ovarian cancer molecular subtypes shows three predominant clusters of gene sets. The first cluster is predominantly composed of proliferation-associated gene sets. The second cluster comprised a mixture of EMT-associated gene sets, gene sets associated with angiogenesis, and with developmental processes. The third is comprised predominantly of gene sets associated with inflammation.

The proliferation cluster of gene sets is strongly associated with poor prognosis in breast cancer overall and ER+ breast cancer subtypes. This supports prior studies demonstrating that proliferation is the strongest factor associated with prognosis in breast cancer overall [15] and in its ER+ molecular subtypes [6]. Interestingly, the proliferation cluster of gene sets shows little association with survival in ER−/HER2− and HER2+ breast cancer and ovarian cancer and its subtypes, and it is the EMT, hypoxia, angiogenesis, and development-associated cluster of gene sets that are associated with poor prognosis in these diseases/subtypes with these pathways showing little association with poor prognosis in ER+ breast cancer. The cluster of immune-related pathways tends to show association with improved prognosis across breast and ovarian cancer and their subtypes (**Figure 10**).

## Discussion

A significant body of work has focused on identifying prognostic signatures in breast cancer. Recently, Venet et al. showed that most random signatures are able to stratify patients into groups that show significantly different survival [15]. This work suggests that more sophisticated and statistically rigorous methods are needed to identify biologically informative gene sets based on observed prognostic associations. Here we describe such a statistical and computational framework (Significance Analysis of Prognostic Signature (SAPS)) to allow robust and biologically informative prognostic gene sets to be identified in disease. The basic premise of SAPS is that in order for a candidate gene set's association with prognosis to be used to imply its biological significance, the gene set must satisfy three conditions.

First, the gene set should cluster patients into prognostically variable groups. The p value generated from this analysis is the standard *P_pure_*, which has been frequently used in the literature to indicate a gene set's clinical and biological relevance for a particular disease. A key insight of the SAPS method (building on the work of Venet et al. [15]) is that clinical utility and biological relevance of a gene set are two very different properties, necessitating distinct statistical tests. The *P_pure_* assesses the statistical significance of survival differences observed between two groups of patients stratified using a candidate gene set, and thus this test provides insight into the potential clinical utility of a gene set for stratifying patients into prognostically variable groups; however, this statistical test provides no information to compare the prognostic performance of the candidate gene set with randomly generated (“biologically null”) gene sets. We believe that it is essential for a candidate prognostic gene set to not only stratify patients into prognostically variable groups, but to do so in a way that is significantly superior to a random gene set of similar size. Therefore, the second condition of the SAPS method is that a gene set must stratify patients significantly more effectively than a random gene set. This analysis produces the *P_random_*. The *P_random_* directly compares the prognostic association of a candidate gene set with the prognostic association of “biologically null” random gene sets. Lastly, to avoid selecting a gene set that is linked to prognosis solely by the unsupervised k-means clustering procedure, the SAPS procedure additionally requires a prognostic gene set to be enriched for genes that show strong univariate associations with prognosis. Therefore, the third condition of the SAPS method is that a candidate gene set should achieve a statistically significant *P_enrichment_*, which is a measure of the statistical significance of a candidate gene set's enrichment with genes showing strong univariate prognostic associations. Our results in breast and ovarian cancer and their molecular subtypes demonstrate that the *P_enrichment_* shows only moderate overall correlation with the *P_pure_* and *P_random_* (range Spearman rho = (0.23–0.35), median Spearman rho = 0.30)) and there is only moderate overlap between gene sets identified at a raw p value of 0.05 by *P_pure_*, *P_random_*, and *P_enrichment_* (Figures 2A–9A). These data suggest that the *P_enrichment_* provides useful additional information to the *P_pure_* and *P_random_* and allows prioritization of gene sets that are enriched for genes showing strong univariate prognostic associations.

Summarizing these three distinct statistical tests into a single score is a difficult task as they were each generated using different methods and they test different hypotheses. We chose to use the maximum as the summary function (as opposed to a median or average, for example), as the maximum is a conservative summary measure and it is easily interpretable. It is important to note that the SAPS method provides users with the SAPS*_score_* as well as all 3 component *P* values (and the 3 component q-values corrected for multiple hypotheses to control the FDR), and therefore the user can choose to use the SAPS*_score_* or to focus on a particular SAPS component, as desired for the specific experimental question being evaluated. Importantly, the SAPS method also performs a permutation-test to estimate the statistical significance of gene set's *SAPS_score_*.

To test the utility of SAPS in providing insight into prognostic pathways in cancer, we performed a systematic, comprehensive, and well-powered analysis of prognostic gene signatures in breast and ovarian cancers and their molecular subtypes. This represents the largest meta-analysis of subtype-specific prognostic pathways ever performed in these malignancies. The analysis identified new prognostic gene sets in breast and ovarian cancer molecular subtypes, and demonstrated significant variability in prognostic associations across the diseases and their subtypes.

We find that proliferation drives prognosis in ER+ breast cancer, while pathways related to hypoxia, angiogenesis, development, and expression of extracellular matrix-associated proteins drive prognosis in ER−/HER2− and HER2+ breast cancer and ovarian cancer. We see an association of immune-related pathways with improved prognosis across all subtypes of breast and ovarian cancers. Our analysis demonstrates that prognostic pathways in HER2+ and ER−/HER2− breast cancer are far more similar to prognostic pathways in angiogenic and non-angiogenic ovarian cancer than to prognostic pathways in ER+ breast cancer. This finding parallels the recent identification of similar genomic alterations in ovarian cancer and basal-like (ER−/HER2−) breast cancer [31].

These results demonstrate the importance of performing subtype-specific analyses to gain insight into the factors driving biology in cancer molecular subtypes. If molecular subtype is not accounted for, prognostic gene sets identified in breast cancer are strongly associated with proliferation [15]; however, when subtype is accounted for, significant and highly distinct pathways (showing no significant association with proliferation) are identified as driving prognosis in ER− breast cancer subtypes. Overall, these data show the utility of performing subtype-specific analyses and using SAPS to test the significance of prognostic pathways. Furthermore, our data suggest that ER− breast cancer subtypes and ovarian cancer may share common therapeutic targets, and future work should address this hypothesis.

In summary, we believe SAPS will be widely useful for the identification of prognostic and predictive biomarkers from clinically annotated genomic data. The method is not specific to gene expression data and can be directly applied to other genomic data types. In the future, we believe that prior to reporting a prognostic gene set, researchers should be encouraged (and perhaps required) to apply the SAPS (or a related) method to ensure that their candidate prognostic gene set is significantly enriched for prognostic genes and stratifies patients into prognostic groups significantly better than the stratification obtained by random gene sets.

## Methods

### Breast Cancer Datasets

Data-sets were provided as Supplemental Material in Haibe-Kains et al. [20]. Our analysis included 19 datasets with survival data (total n = 3832) (****).

### Ovarian Cancer Datasets

Data-sets were provided as Supplemental Material in Bentink et al. [21]. Our analysis included 1735 ovarian cancer patients for whom overall survival data were available (**Table S2**).

### Molecular Subtype Classification

For breast cancer, the SCMGENE model [20] was used in the R/Bioconductor *genefu* package [22] to stratify patients into four molecular subtypes: ER+/HER2− low proliferation, ER+/HER2− high proliferation, ER−/HER2− and HER2+. In the ovarian datasets we used ovcAngiogenic model [21] as implemented in *genefu*.

### Creation of Meta-Data Sets

For genes with multiple probes, we selected the probe with the highest variance. We tested two procedures for merging of data: subtype-specific scaling, and traditional (non subtype-specific scaling) (as described in “Data-Scaling and Merging” portion of the manuscript). We excluded genes and cases with more than 50% of data missing. From these reduced data matrices, we imputed missing values using the *impute* package in R [32]. These pre-processed meta-data sets are included as Supporting Information in Dataset S1 for both breast and ovarian cancer using subtype-specific and traditional scaling.

### Gene Sets

Gene sets from the Molecular Signatures Database (MSigDB) [17] (http://www.broadinstitute.org/gsea/msigdb/collections.jsp) (“molsigdb.v3.0.entrez.gmt”). Analyses were limited to gene sets of size greater than 1 and less than or equal to 250 genes.

### Application of the Significance Analysis of Prognostic Signatures (SAPS) Procedure and Visualization of SAPS P Values

The SAPS procedure is described in “Significance Analysis of Prognostic Signatures (SAPS)” portion of the manuscript. Briefly, for a candidate gene set, SAPS generates 3 component p-values: *P_pure_*, *P_random_*, and *P_enrichment_*. The *SAPS_score_* is the maximum of these values. The *P_pure_* is the standard log-rank p value, computed by performing K-means clustering with a k of 2 and assessing the statistical significance of the survival difference between the 2 resulting clusters, implemented using the survdiff function in the R package *survival* and extracting the chi-square statistic for a test of equality of the 2 survival curves. To compute the *P_random_*, we generate a distribution of *P_pure_* from “random” gene sets (we used 10000 random gene sets for a sequence of 8 gene set sizes ranging from 5 to 250), and we calculate the proportion of random gene sets of a similar size to the candidate gene sets that achieve a *P_pure_* at least as significant as the true *P_pure_*. To compute the *P_enrichment_*, we generate “.rnk” files that include each gene and its concordance index for survival, implemented with the function concordance.index in the *survcomp* R package. These “.rnk” files are used in a pre-ranked GSEA analysis implemented with the executable jar file gsea2-2.07 (which is downloadable from: http://www.broadinstitute.org/gsea/downloads.jsp). In our analyses, we set a maximum gene set size of 250 and used default GSEA parameters. The *SAPS_score_* for each candidate gene set is then computed as the negative log_10_ of the maximum of the (*P_pure_*, *P_random_*, and *P_enrichment_*) times the direction of the association (positive or negative). The statistical significance of the *SAPS_score_* is determined by permutation-testing. Specifically, in our experiments, we performed 10000 permutations of the gene labels for each of the sequence of 8 of gene set sizes ranging from 5 to 250. We performed the full SAPS procedure for each of the 80000 permuted gene sets and we generated a null distribution of 10000 *SAPS_scores_* for each of the 8 gene set sizes. The *SAPS_p-value_* was computed as the proportion of permuted gene sets of a similar size to the candidate gene set that achieved at least as extreme a *SAPS_score_*. The *SAPS_p-values_* were then converted to *SAPS_q-values_* using the method of Benjamini and Hochberg [19].

### Hierarchical Clustering

Hierarchical clustering was performed on the SAPS scores for breast and ovarian cancer molecular subtypes. Hierarchical clustering was performed with one minus Spearman rank correlation as the distance metric and complete linkage, using the Cluster 3.0 package (http://bonsai.hgc.jp/~mdehoon/software/cluster/). Clustering results were visualized using Java TreeView (http://jtreeview.sourceforge.net/). The Java TreeView files used to generate the Heatmap in Figure 10 are provided in the Supplementary Information (“BreastOvary_HC.zip”).

An R script and R workspaces for running SAPS on the breast and ovarian cancer meta-data sets and generating Scatterplots and Venn Diagrams of the SAPS P-Values (including all figures from our analyses) are included in in Dataset S1 (http://dx.doi.org/10.5061/dryad.mk471). The Venn diagrams were generated with the *Vennerable* package in R.

## Supporting Information

Dataset S1 Supporting information data files, R scripts, and R workspaces. Data deposited in the Dryad repository: http://dx.doi.org/10.5061/dryad.mk471.
(DOCX)

Table S1 Breast cancer datasets.
(DOCX)

Table S2 Ovarian cancer datasets.
(DOCX)

Table S3 This excel workbook presents the results of the SAPS analyses in breast cancer. The first column is the molsigdb gene set id. The second column is gene set size. The third column is the *SAPS_q-value_*. The fourth column is the *SAPS_score_*. The fifth through seventh columns are the raw *P_pure_*, *P_random_*, and *P_enrichment_*, respectively. The eighth through 10^th^ columns are the q-values associated with the *P_pure_*, *P_random_*, and *P_enrichment_*. The final column indicates the direction of the prognostic association. Each disease or disease subtype analysis is on one sheet of the workbook.
(XLS)

Table S4 This excel workbook presents the results of the SAPS analyses in ovarian cancer. The first column is the molsigdb gene set id. The second column is gene set size. The third column is the *SAPS_q-value_*. The fourth column is the *SAPS_score_*. The fifth through seventh columns are the raw *P_pure_*, *P_random_*, and *P_enrichment_*, respectively. The eighth through 10^th^ columns are the q-values associated with the *P_pure_*, *P_random_*, and *P_enrichment_*. The final column indicates the direction of the prognostic association. Each disease or disease subtype analysis is on one sheet of the workbook.
(XLS)
