# Supplementary material for: Significance Analysis of Prognostic Signatures
Source: PLoS Comput Biol. 2013 Jan 24;9(1):e1002875. doi: 10.1371/journal.pcbi.1002875 (PMC3554539; doi:10.1371/journal.pcbi.1002875)
Supplement: Dataset S1 — Supporting information data files, R scripts, and R workspaces. Data deposited in the Dryad repository: http://dx.doi.org/10.5061/dryad.mk471. (DOCX) [file pcbi.1002875.s001.docx]

**Dataset S1­­ —** Supporting information data files, R scripts, and R workspaces. Data deposited in the Dryad repository: <http://dx.doi.org/10.5061/dryad.mk471>

The **Dataset S1** files are described below (Additional description of the files and ReadMe files are provided at datadryad.org).

*saps.R* – This R script provides R commands for loading data, applying the SAPS method, and generating the SAPS p values. The script is interactive, and the user must specify the working directory, and if the analysis is on the ovarian or breast data.

runSAPSonPermutedData.R – This R script generates the P_pure, P_random, and P_enrichment on random gene sets.

computeSAPS.Permute.PValue.R – This script generates permutation-based p and q values for the SAPSscores obtained in breast and ovarian cancer.

sapsFigures.R – This R script generates the figures, tables, and file used for clustering

*Breast.RData* – This R-workspace contains the objects: dat, dat.st, event, st, and time.

| Breast.RData |  |
| --- | --- |
| dat | Data scaled within each dataset without knowledge of subtype. Data from all data-sets merged into this object, which contains expression data on 2731 patients for 13091 genes. Patients are in rows, and entrezID’s in columns. |
| dat.st | Data scaled within molecular subtype within each dataset. Data from all data-sets merged into this object, which contains expression data on 2731 patients for 13091 genes. Patients are in rows, and entrezID’s in columns. |
| time | Time (days) |
| event | Distant metastasis or death |
| st | Molecular subtype defined by SCMGENE |

*Ovary.RData* – This R-workspace contains the objects: dat, dat.st, event, st, and time.

| Ovary.RData |  |
| --- | --- |
| dat | Data scaled within each dataset without knowledge of subtype. Data from all data-sets merged into this object, which contains expression data on 1670 patients for 11247 genes. Patients are in rows, and entrezID’s in columns. |
| dat.st | Data scaled within molecular subtype within each dataset. Data from all data-sets merged into this object, which contains expression data on 1670 patients for 11247 genes. Patients are in rows, and entrezID’s in columns. |
| time | Time (days) |
| event | Death |
| st | Molecular subtype defined by SCMGENE |

*BreastOutput_TradScaled.RData*– This R-workspace contains the objects: allPs, allPs.adj, sumTable.

| BreastOutput_TradScaled.RData |  |
| --- | --- |
| allPs | Contains raw p values for 5320 genesets in molsigdb.v3.0. The columns indicate the type of p value (P_pure, P_random, P_gsea) and the analysis that generated the p value (Global = “Global analysis”, ER_H = “ER+ High proliferation”, ER_L = “ER+ Low proliferation”, H2 = “HER2+”,TN = “ER-/HER2-“). These p values were generated on the traditional (non-subtype specific) scaled data. |
| allPs.adj | Matrix contains the adjusted p values using the method of Benajmini and Hochberg on the traditional (non-subtype specific) scaled data. |

*BreastOutput_SubScaled.RData*– This R-workspace contains the objects: allPs, allPs.adj, sumTable.

| BreastOutput_SubScaled.RData |  |
| --- | --- |
| allPs | Contains raw p values for 5320 genesets in molsigdb.v3.0. The columns indicate the type of p value (P_pure, P_random, P_gsea) and the analysis that generated the p value (Global = “Global analysis”, ER_H = “ER+ High proliferation”, ER_L = “ER+ Low proliferation”, H2 = “HER2+”,TN = “ER-/HER2-“). These p values were generated on the subtype-specific scaled data. |
| allPs.adj | Matrix contains the adjusted p values using the method of Benajmini and Hochberg on the subtype-specific scaled data. |

*OvaryOutput_TradScaled.RData*– This R-workspace contains the objects: allPs, allPs.adj, sumTable.

| OvaryOutput_TradScaled.RData |  |
| --- | --- |
| allPs | Contains raw p values for 5355 genesets in molsigdb.v3.0. The columns indicate the type of p value (P_pure, P_random, P_gsea) and the analysis that generated the p value (Global = “Global analysis”, Angio= “Angiogenic subtype” , Non-Angio = “Non-angiogenic subtype”. These p values were generated on the traditional (non-subtype specific) scaled data. |
| allPs.adj | Matrix contains the adjusted p values using the method of Benajmini and Hochberg on the traditional (non-subtype specific) scaled data. |

*OvaryOutput_SubScaled.RData*– This R-workspace contains the objects: allPs, allPs.adj, sumTable.

| OvaryOutput_SubScaled.RData |  |
| --- | --- |
| allPs | Contains raw p values for 5355 genesets in molsigdb.v3.0. The columns indicate the type of p value (P_pure, P_random, P_gsea) and the analysis that generated the p value (Global = “Global analysis”, Angio= “Angiogenic subtype” , Non-Angio = “Non-angiogenic subtype”. These p values were generated on the subtype-specific scaled data. |
| allPs.adj | Matrix contains the adjusted p values using the method of Benajmini and Hochberg on the subtype-specific scaled data. |

*FinalOutput_Breast.RData contains the results from the subtype-specific analysis in breast cancer, including the results of the permutation-based procedure to compute p values and q values for the SAPSscores.*

| FinalOutput_Breast.RData |  |
| --- | --- |
| allPs | Contains raw p values for 5320 genesets in molsigdb.v3.0. The columns indicate the type of p value (P_pure, P_random, P_gsea) and the analysis that generated the p value (Global = “Global analysis”, ER_H = “ER+ High proliferation”, ER_L = “ER+ Low proliferation”, H2 = “HER2+”,TN = “ER-/HER2-“). These p values were generated on the subtype-specific scaled data. |
| allPs.adj | Matrix contains the adjusted p values using the method of Benajmini and Hochberg on the subtype-specific scaled data. |
| saps.p | Permutation-based p value for each gene set in molsigdb generated on the subtype-specific scaled data |
| saps.p.adj | Adjusted p value (q-value) to indicate the statistical significance of each gene set’s SAPSScore |
| saps.score | This matrix contains the maximum of each gene set’s raw (P_pure, P_random, P_gsea) |
| saps.score.adj | This matrix contains the maximum of gene set’s adjusted (P_pure, P_random, P_gsea) |
| saps.score.r | Array of dimensions 8 x 10000 x 6. The first dimension is the 8 sizes (from 5 to 250) of the random gene sets. The second dimension is the 10000 permutations. The third dimension is the 6 breast cancer analyses performed (Global and the 5 subtypes). Each cell in the array contains the *SAPS_Score_* obtained with a permuted gene set. |

*FinalOutput_Ovary.RData contains the results from the traditional scaled data set in ovarian cancer, including the results of the permutation-based procedure to compute p values and q values for the SAPS_Scores_.*

| FinalOutput_Breast.RData |  |
| --- | --- |
| allPs | Contains raw p values for 5355 genesets in molsigdb.v3.0. The columns indicate the type of p value (P_pure, P_random, P_gsea) and the analysis that generated the p value (Global = “Global analysis”, Angio= “Angiogenic subtype” , Non-Angio = “Non-angiogenic subtype”. These p values were generated on the traditional (non-subtype specific) scaled data. |
| allPs.adj | Matrix contains the adjusted p values using the method of Benajmini and Hochberg on the traditional (non-subtype specific) scaled data. |
| saps.p | Permutation-based p value for each gene set in molsigdb generated on the traditional scaled data |
| saps.p.adj | Adjusted p value (q-value) to indicate the statistical significance of each gene set’s *SAPS_Score_* |
| saps.score | This matrix contains the maximum of each gene set’s raw (P_pure, P_random, P_gsea) |
| saps.score.adj | This matrix contains the maximum of gene set’s adjusted (P_pure, P_random, P_gsea) |
| saps.score.r | Array of dimensions 8 x 10000 x 3. The first dimension is the 8 sizes (from 5 to 250) of the random gene sets. The second dimension is the 10000 permutations. The third dimension is the 3 ovarian cancer analyses performed (Global and the 2 subtypes). Each cell in the array contains the *SAPS_Score_* obtained with a permuted gene set. |

*Breast.Ps.OnPermutedData.RData* contains the results of performing SAPS using permuted gene sets on the breast data

| Breast.Ps.OnPermutedData.RData |  |
| --- | --- |
| P_enrich, p_pure,p_rand | 8 x 10000 x 6 arrays with P_enrich,P_pure, and P_random p values from permuted gene sets |

*Ovary.Ps.OnPermutedData.RData* contains the results of performing SAPS using permuted gene sets on the ovarian data

| Ovary.Ps.OnPermutedData.RData |  |
| --- | --- |
| P_enrich, p_pure,p_rand | 8 x 10000 x 6 arrays with P_enrich,P_pure, and P_random p values from permuted gene sets |

BreastSubtypeSpecScaleRankDir contains the ranked gene lists of concordance indices used to perform the GSEA in breast cancer

OvaryTradScaleRankDir contains the ranked gene lists used of concordance indices to perform the GSEA in ovarian cancer

*BreastOvary_HCv2* – This directory contains files to generate Figure 10 (Hierarchical clustering of breast and ovarian cancer subtypes based on SAPS scores) using JavaTreeView (<http://jtreeview.sourceforge.net/>)

*molsigdb.v3.0.entrezForR –* This file is used to read the molsigdb.v3.0 gene sets into R.

*GSEA Results: The GSEA results for each cancer subtype are presented in the directories: Breast_Global, Breast_ERHigh, Breast_ERLow,Breast_ERNegHer2Neg,Breast_Her2, Ovary_Global, Ovary_Angio, Ovary_NonAngio*. These analyses were performed to generate the P_enrichment as part of the SAPS Procedure. Results can be visualized by clicking the index.html file in each directory.
