# Supplementary material for: Significance Analysis of Prognostic Signatures
Source: PLoS Comput Biol. 2013 Jan 24;9(1):e1002875. doi: 10.1371/journal.pcbi.1002875 (PMC3554539; doi:10.1371/journal.pcbi.1002875)
Supplement: Table S1 — Breast cancer datasets. (DOCX) [file pcbi.1002875.s002.docx]

**Supporting Information Table 1**

| **Dataset** | **Microarray technology** | **Survival data** | **No. of patients** | **Source** | **Reference** |
| --- | --- | --- | --- | --- | --- |
| VDX | Affymetrix HGU | RFS, DMFS | 688 | GEO: GSE2034/GSE5327 | [[1](#_ENREF_1), [2](#_ENREF_2)] |
| NKI | Agilent | RFS, DMFS,  OS | 319 | Rosetta Inpharmatics | [[3](#_ENREF_3), [4](#_ENREF_4)] |
| UCSF | in-house cDNA | DNFS, RFS,  OS | 162 | Authors’ website | [[5](#_ENREF_5), [6](#_ENREF_6)] |
| STNO2 | in-house cDNA | RFS, OS | 118 | SMD | [[7](#_ENREF_7)] |
| NCI | in-house cDNA | RFS | 99 | Authors’ website | [[8](#_ENREF_8)] |
| MSK | Affymetrix HGU | DMFS | 82 | GEO: GSE2603 | [[9](#_ENREF_9)] |
| UPP | Affymetrix HGU | RFS | 236 | GEO: GSE3494 | [[10](#_ENREF_10)] |
| STK | Affymetrix HGU | RFS | 159 | GEO: GSE1456 | [[11](#_ENREF_11)] |
| UNT | Affymetrix HGU | RFS, DMFS | 133 | GEO: GSE2990 | [[12](#_ENREF_12), [13](#_ENREF_13)] |
| UNC4 | Agilent | RFS, OS | 241 | UNC DB | [[14](#_ENREF_14)] |
| CAL | Affymetrix HGU | RFS, DMFS,  OS | 117 | AE: E-TABM-158 | [[16](#_ENREF_16)] |
| TRANSBIG | Affymetrix HGU | RFS, DMFS,  OS | 198 | GEO: GSE7390 | [[17](#_ENREF_17)] |
| MAINZ | Affymetrix HGU | DMFS | 200 | GEO: GSE11121 | [[18](#_ENREF_18)] |
| EMC2 | Affymetrix HGU | DMFS | 204 | GEO: GSE12276 | [[19](#_ENREF_19)] |
| DFHCC | Affymetrix HGU | DMFS | 115 | GEO: GSE19615 | [[20](#_ENREF_20)] |
| TAM | Affymetrix HGU | DMFS, RFS | 242 | GEO: GSE6532/GSE9195 | [[21](#_ENREF_21)] |
| MDA5 | Affymetrix HGU | DMFS | 298 | GEO: GSE17705 | [[22](#_ENREF_22)] |
| VDX3 | Affymetrix HGU | DMFS | 136 | GEO: GSE12093 | [[23](#_ENREF_23)] |
| PNC | Affymetrix HGU | OS | 85 |  |  |

*Microarray datasets of unique breast cancer patients (3832) used in this study were retrieved from authors’ websites, Gene Expression Omnibus (GEO; http://www.ncbi.nlm.nih.gov/geo/), ArrayExpress (AE; http://www.ebi.ac.uk/arrayexpress/), Stanford Microarray Database (SMD; http://smd.stanford.edu/), MD Anderson Cancer Center Microarray database (MDACC DB; http://bioinformatics.mdanderson.org/pubdata.html), University of North Carolina database (UNC DB; https://genome.unc.edu/), and Rosetta Inpharmatics (<http://www.rosettabio.com/>). Each dataset was assigned a short acronym and an instance number if several datasets were published by the same institution or consortium: EXPO: expression project for oncology, large dataset of microarray data published by the International Genomics Consortium (United States); VDX: Veridex (The Netherlands); NKI: National Kanker Instituut (The Netherlands); UCSF: University of California, San Francisco (United States); STNO: Stanford/Norway (United States and Norway); NCI: National Cancer Institute (United States); MSK Memorial Sloan-Kettering (United States); UPP: Uppsala hospital (Sweden); STK: Stockholm. Karolinska university hospital (Sweden); UNT: cohort of untreated breast cancer patients from the Oxford Radcliffe (United Kingdom) and Karolinska (Sweden) hospitals; UNC: University of North Carolina (United States); DUKE: Duke university hospital (United States); CAL: dataset of breast cancer patients from the University of California, San Francisco and the California Pacific Medical Center (United States); TRANSBIG: dataset collected by the TransBIG consortium (Europe); MAINZ: Mainz hospital (Germany); LUND: Lund University Hospital (Sweden); FNCLCC: Fédération Nationale des Centres de Lutte contre le Cancer (France); MDA: MD Anderson Cancer Centter (United States); EMC: Erasmus Medical Center (The Netherlands); MUG: Medical University of Graz (Austria); NCCS: National Cancer Centre of Singapore (Singapore); MCCC: Peter MacCallum Cancer Centre (Australia); KOO: Koo Foundation Sun Yat-Sen Cancer Centre (Taiwan); EORTC10994: Trial number 10994 from the European Organization for Research and Treatment of Cancer Breast Cancer; (Europe) HLP: University Hospital La Paz (Spain); DFHCC: Dana-Farber Harvard Cancer Center (United States); MAQC: Microarray quality control consortium (United States); JBI: Jules Bordet Institute (Belgium). These datasets were generated with diverse microarray technologies developed either by Agilent (http://www.genomics.agilent.com), Affymetrix (HGU GeneChips, which include chips HG-U133A, HG-U133B and HG-U133PLUS2, and X3P GeneChip; http://www.affymetrix.com), Swegene (http://www.genomics.agilent.com), Operon (<http://www.operon.com>) or developed in-house (complementary DNA, cDNA, platforms). For most datasets survival data (distant metastasis-free survival [DMFS], relapse-free survival [RFS], and overall survival [OS]) and information regarding the adjuvant treatment (untreated, chemo, hormonal, and heterogeneous standing for no treatment, chemotherapy, hormonal therapy and heterogeneous combination of therapies, respectively) was available, otherwise missing information is referred to as not available (NA). Additional clinical characteristics are provided in Table 2. All untreated patients had surgery, and most of them had radiation therapy, although information is not available for all datasets.
