# Supplementary material for: Significance Analysis of Prognostic Signatures
Source: PLoS Comput Biol. 2013 Jan 24;9(1):e1002875. doi: 10.1371/journal.pcbi.1002875 (PMC3554539; doi:10.1371/journal.pcbi.1002875)
Supplement: Table S2 — Ovarian cancer datasets. (DOCX) [file pcbi.1002875.s003.docx]

**Supporting Information Table 2**

**Supplementary Table 2**: Compendium of microarray datasets of unique ovarian cancer patients

| **Dataset** | **Microarray technology** | **Survival data** | **Treatment** | **No. of patients** | **Number of probes** | **Source** | **Reference** |
| --- | --- | --- | --- | --- | --- | --- | --- |
| DFCI | Ilumina DASL | RFS, OS | Platinum, chemo | 129 | 12,469 | AE: E-MTAB-386 | [[69](#_ENREF_69)] |
| DUKE | Affymetrix HGU | OS | Platinum, chemo | 118 | 22,283 | http://data.cgt.duke.edu/platinum.php | [[70](#_ENREF_70)] |
| FIGO | Agilent G4112A | OS | Platinum, chemo | 110 | 41,000 | GEO: GSE17260 | [[71](#_ENREF_71)] |
| AOCS | Affymetrix HGU | RFS, OS | Platinum, chemo | 285 | 54,675 | GEO: GSE9899 | [[72](#_ENREF_72)] |
| MSKCC | Affymetrix HGU | OS | Platinum, chemo | 185 | 22,283 | GEO: GSE26712 | [[73](#_ENREF_73)] |
| TCGA | Affymetrix HGU | RFS, OS | Platinum, chemo | 510 | 22,283 | http://tcga-data.nci.nih.gov/tcga/tcgaHome2.jsp | [[74](#_ENREF_74)] |
| BIDMC | Affymetrix HG-U95v2 | OS | Platinum, chemo | 53 | 12,625 | GEO: GSE19161 | [[75](#_ENREF_75)] |
| UPENN | Affymetrix HGU | OS | Platinum, chemo | 55 | 54,675 | GEO: GSE19161 | [[76](#_ENREF_76)] |
| TOC | Affymetrix HGU | OS | Platinum, chemo | 80 | 22,283 | GEO: GSE14764 | [[77](#_ENREF_77)] |
| UMCG | Operon human v3 35K | OS | Platinum, chemo | 157 | 15,909 | GEO: GSE13876 | [[78](#_ENREF_78)] |
| BWH | Affymetrix HGU | OS | Platinum, chemo | 53 | 54,675 | GEO: GSE18520 | [[79](#_ENREF_79)] |

*Microarray datasets of unique breast cancer patients (5715) used in this study were retrieved from authors’ websites, Gene Expression Omnibus (GEO; http://www.ncbi.nlm.nih.gov/geo/), ArrayExpress (AE; <http://www.ebi.ac.uk/arrayexpress/>). Each dataset was assigned a short acronym and an instance number if several datasets were published by the same institution or consortium: DFCI: Dana-Farber Cancer Institute (United States); DUKE: Duke university hospital (United States); FIGO: International Federation of Gynecology and Obstetrics (Japan); AOCS: Australian Ovarian Cancer Study (Australia); MSKCC: Memorial Sloan-Kettering Cancer Center (United States); TCGA: The Cancer Genome Atlas (United States); BIDMC: Beth Israel Deaconess Medical Center (United States); UPENN: University of Pennsylvania (United States); TOC: tumour bank ovarian cancer (Europe); UMCG: University Medical Center Groningen (The Netherlands); BWH: Brigham and Women’s Hospital (United States). These datasets were generated with diverse microarray technologies developed either by Agilent (http://www.genomics.agilent.com), Affymetrix (HGU GeneChips, which include chips HG-U133A, HG-U133B and HG-U133PLUS2 GeneChips; http://www.affymetrix.com), or Operon (<http://www.operon.com>). For most datasets survival data (relapse-free survival [RFS], and overall survival [OS]) and information regarding the adjuvant treatment (platinum, chemo standing for platinum and chemotherapy respectively) was available, otherwise missing information is referred to as not available (NA).
